# Proteome-Wide Analysis of *Trypanosoma cruzi* Exponential and Stationary Growth Phases Reveals a Subcellular Compartment-Specific Regulation

**DOI:** 10.3390/genes9080413

**Published:** 2018-08-15

**Authors:** Carla Cristi Avila, Simon Ngao Mule, Livia Rosa-Fernandes, Rosa Viner, María Julia Barisón, André Guillherme Costa-Martins, Gilberto Santos de Oliveira, Marta Maria Geraldes Teixeira, Claudio Romero Farias Marinho, Ariel Mariano Silber, Giuseppe Palmisano

**Affiliations:** 1 Department of Parasitology, Institute of Biomedical Sciences, University of Sao Paulo, 05508-900 Sao Paulo, Brazil; carla.cristi@gmail.com (C.C.A.); ngaosimon@gmail.com (S.N.M.); liviarosa.f@gmail.com (L.R.-F.); mjbarison@gmail.com (M.J.B.); andreguilherme@usp.br (A.G.C.-M.); gilberto_so@outlook.com (G.S.d.O.); mmgteix@icb.usp.br (M.M.G.T.); crfmarinho@gmail.com (C.R.F.M.); asilber@usp.br (A.M.S.); 2 Department of Biochemistry and Molecular Biology, University of Southern Denmark, 5230 Odense, Denmark; 3 Thermo Fisher Scientific, San Jose, CA 95134, USA; rosa.viner@thermofisher.com

**Keywords:** *Trypanosoma cruzi*, metacyclogenesis, exponential and stationary phases, quantitative proteomics, Gim5a, *N*-acetyltransferases, protein N-terminal acetylation, autophagy

## Abstract

*Trypanosoma cruzi*, the etiologic agent of Chagas disease, cycles through different life stages characterized by defined molecular traits associated with the proliferative or differentiation state. In particular, *T. cruzi* epimastigotes are the replicative forms that colonize the intestine of the Triatomine insect vector before entering the stationary phase that is crucial for differentiation into metacyclic trypomastigotes, which are the infective forms of mammalian hosts. The transition from proliferative exponential phase to quiescent stationary phase represents an important step that recapitulates the early molecular events of metacyclogenesis, opening new possibilities for understanding this process. In this study, we report a quantitative shotgun proteomic analysis of the *T. cruzi* epimastigote in the exponential and stationary growth phases. More than 3000 proteins were detected and quantified, highlighting the regulation of proteins involved in different subcellular compartments. Ribosomal proteins were upregulated in the exponential phase, supporting the higher replication rate of this growth phase. Autophagy-related proteins were upregulated in the stationary growth phase, indicating the onset of the metacyclogenesis process. Moreover, this study reports the regulation of N-terminally acetylated proteins during growth phase transitioning, adding a new layer of regulation to this process. Taken together, this study reports a proteome-wide rewiring during *T. cruzi* transit from the replicative exponential phase to the stationary growth phase, which is the preparatory phase for differentiation.

## 1. Introduction

Cell proliferation is controlled by a series of environmental factors such as temperature, population density due to the presence of critical quantities of quorum-sensing molecules and specific growth factors, osmolarity, pH, the redox state of the environment, and the availability of essential nutrients [1,2]. Cells are usually able to sense all these factors and orchestrate a variety of responses. As a result, cells initially face a binary decision: proliferate or enter into a specialized non-dividing resting state, known as stationary/quiescent phase or G0 [3]. For example, it is well demonstrated that under a severe metabolic stress condition known as starvation, yeasts arrest their cell cycle and then enter into a G0/quiescence state that promotes persistence and survival [3]. Usually, this change is concomitant with deep modification of the cells’ structures that can yield resistance structures such as cysts or spores [4,5]. Besides cell cycle arrest, other changes are observed during transitioning from proliferating to the quiescent phase, such as changes in their mass and volume, transcription, mRNA degradation, and translation, resulting in a different protein expression pattern. Additionally, the autophagy process is frequently induced in the stationary phase [6]. As a result, quiescent cells become more thermotolerant and osmotolerant than their proliferating counterparts [7].

*Trypanosoma cruzi*, the etiologic agent of Chagas disease, cycles through different life stages that harbor intricate molecular features that are needed in order to survive into the alimentary tract of the invertebrate vector, and the bloodstream and the intracellular environment of the vertebrate host [8]. In particular, epimastigote forms are replicative in the Triatominae insects, colonizing their digestive tract. An active proliferation is needed for establishing the infection in the insect host, while entering in the stationary phase is a condition for its survival and differentiation into metacyclic trypomastigotes, which is the stage that is able to initiate the infectious process in mammals. Thus, a refined regulation of the cell physiology in these states is a conditio sine qua non for the parasite to complete its natural life cycle [9].

One of the most critical environmental factors affecting the proliferative/quiescent state of cells is the availability of nutrients. In the case of *T. cruzi* epimastigotes, it was shown that the progressive changes of nutrients availability in in vitro cultures lead initially to an adaptive metabolic switch from a metabolism based on carbohydrates consumption to a metabolism based on amino acids consumption [10,11]. Under metabolites exhaustion, *T. cruzi* epimastigotes undergo cell cycle arrest, which is considered a trigger for its differentiation to metacyclic forms [12]. These forms are responsible for infecting the vertebrate host, as shown by a seminal work from Camargo [13]. Due to that, *T. cruzi* in the stationary phase could recapitulate the early molecular events of metacyclogenesis, and can be considered as an onset for differentiation, opening new possibilities to understand the early events of metacyclogenesis. Several efforts have been made to understand the molecular events underlining this process. Parodi-Talice et al. used two-dimensional (2D) gels in combination with matrix-assisted laser desorption/ionization-time-of-flight (MALDI-TOF) analysis to identify proteins involved in the metacyclogenesis [14]. *T. cruzi* Dm28c strain epimastigotes were cultured in liver infusion tryptose (LIT) and collected at the exponential phase. Subsequently, the cells were submitted to nutritional stress in triatomine artificial urine (TAU) medium for 2 h before starting the differentiation using the TAU medium supplemented with glucose, proline, glutamate and aspartate (TAU3AAG) for 3 h and 24 h. Adherent differentiating epimastigotes were collected, and metacyclic trypomastigotes were purified. Almost 50% of the detected protein spots were unique in the different conditions showing a global proteome remodeling during metacyclogenesis. Forty-three proteins were identified and assigned to antioxidant response, protein biosynthesis, and cytoskeleton organization. Interestingly, several proteoforms displayed a differential modulation during metacyclogenesis, indicating the dysregulation of post-translational modification processes. Another study from Godoy et al. applied quantitative shotgun proteomics to dissect the metacyclogenesis process [15]. *T. cruzi* epimastigotes from the Dm28c strain were initially cultured for 5 days in LIT medium, and then submitted to nutritional stress by incubation in TAU medium. After 30 min and 2 h, the parasites were collected before being diluted into TAU3AAG medium, and the adhered parasites were collected after 12 h [16,17]. Metacyclic trypomastigotes were purified by diethylaminoethylcellulose (DEAE–cellulose) chromatography. Quantitative shotgun proteomics analysis allowed the identification of proteins involved in this process. The majority of changes were seen when comparing the metacyclic trypomastigotes with the epimastigotes. Recently, Amorim et al. analyzed the phosphoproteome of the adherent population of *T. cruzi* parasites during metacyclogenesis [18]. In particular, *T. cruzi* epimastigotes from the Dm28c strain were subjected to nutritional stress in TAU medium for 2 h, and then differentiated into the metacyclic trypomastigotes from 6 h to 96 h. The phosphoproteome analysis revealed the regulation of 260 phosphosites associated to a global modulation of kinases and phosphatases during metacyclogenesis. Barisón et al. have studied the metabolic state of *T. cruzi* in the exponential and stationary phases using a targeted metabolomics approach [11]. The metabolic shift from glucose to amino acids consumption was shown in the stationary phase, demonstrating an adaptation of the parasite to nutritional stress conditions.

In this work, we applied a comprehensive quantitative proteomics strategy based on high resolution and accuracy mass spectrometry (MS) to identify the molecular events regulated during transitioning from exponential to stationary growth phases in *T. cruzi*. More than 3000 proteins were detected and quantified in the two phases covering several cellular compartments and biological functions. In particular, ribosomal proteins were massively upregulated in the exponential phase, while the calreticulin—the protein localized in the endoplasmic reticulum—was detected upregulated in the stationary phase. The regulated proteins were associated to metabolic processes and membrane transporters, indicating a proteome-wide rewiring during the transitioning between the two phases. Metabolic proteins such as proline dehydrogenase and malate dehydrogenase were upregulated in the stationary and exponential phases, respectively. Membrane proteins such as ADP/ATP mitochondrial carrier protein was upregulated in the exponential phase, while amastin and Gim5 were upregulated in the stationary phase. Interestingly, the uncharacterized proteins upregulated in the stationary phase were enriched in the autophagy protein Apg6 domain. This domain is associated to the autophagosome formation, which is an important process activated during metacyclogenesis. On the contrary, the WD40 domain G-beta (Gβ) repeat was upregulated in the exponential phase uncharacterized proteins and is associated with cell division and gene transcription. Moreover, this study reports the regulation of N-terminally acetylated proteins during growth phase transitioning. Taken together, the metabolic pressure induced by nutrient starvation remodels the proteome of *T. cruzi* from the exponential to the stationary phase, revealing novel targets for early differentiation events.

## 2. Materials and Methods

### 2.1. Parasites and Sample Preparation

**Figure 1:**
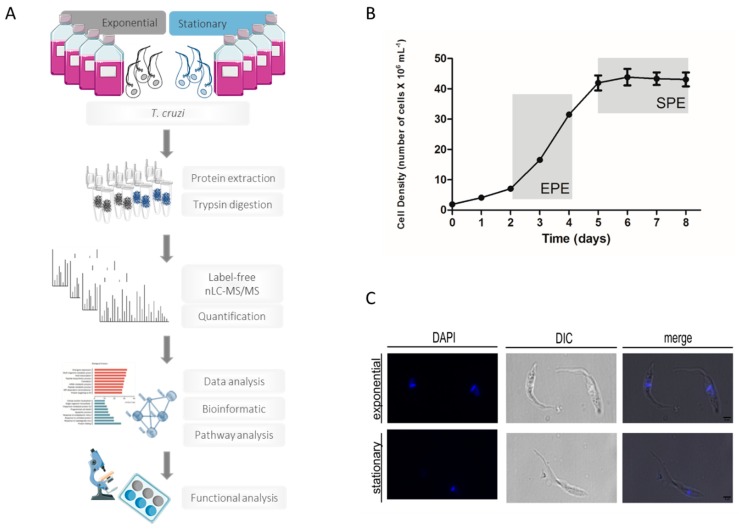
Mass spectrometry-based proteomics approach to assess the regulation of proteins during transitioning from exponential to stationary phase in *Trypanosoma cruzi* cells. (**A**) Proteomics workflow for identification and quantification of regulated proteins in *T. cruzi* during growth phase transitioning. *T. cruzi* epimastigote cells were taken during the exponential and stationary phase before protein extraction and tryptic digestion. Tryptic peptides were analyzed by nano-liquid chromatography tandem mass spectrometry (nLC-MS/MS) analysis. After data and bioinformatic analysis combined with functional studies, this approach made possible the assessment of protein regulation during *T. cruzi* exponential to stationary phase transitioning. (**B**) Epimastigotes growth curve presenting typical exponential and stationary phases with physiological differences. Optic microscopy representative images show the different shape of the exponential and stationary phases of the parasite. (**C**) Microscopic analysis of *T. cruzi* epimastigotes at the exponential and stationary phases using phase-contrast microscope and DAPI staining. DIC: differential interference contrast. Scale bar: 4 μm.

*T. cruzi* CL strain clone 14 epimastigotes [11,19,20] were grown in LIT medium supplemented with 10% fetal calf serum (FCS) at 28 °C [13]. Exponential phase epimastigotes (Exp) were obtained from a 24 h culture starting at 2.5 × 10^7^ parasites/mL (Figure 1). Stationary phase epimastigotes (St) were obtained from an exponential culture at 5.0 × 10^6^ parasites/mL and maintained for four days without media change. Parasites were harvested by centrifugation at 4000 rpm for 10 min and washed twice with cold PBS (137 mM of NaCl, 2.7 mM of KCl, 10 mM of Na_2_HPO_4_, 1.8 mM of KH_2_PO_4_, pH 7.4). The classification of the *T. cruzi* CL-14 strain was performed by gene sequencing [21]. It is important to highlight that *T. cruzi* cells in the stationary phase were epimastigotes, since metacyclic trypomastigotes started to appear later at seven days post-culture and constituted a minor portion (less than 7%) of the total cells.

### 2.2. Protein Extraction and Digestion

The samples were separately resuspended in PBS extraction buffer containing 8 M of urea, 10 mM of dithiothreitol (DTT), benzonase, and a protease inhibitor cocktail (Sigma-Aldrich, St. Louis, MO, USA). Protein concentrations were determined by the Qubit fluorimetric detection method. The proteins were diluted in water to a concentration of 1 of nmol/μL, and reduced by the addition of dithiothreitol (DTT) to a final concentration of 10 mM and incubation for 30 min. The proteins were alkylated prior to digestion by the addition of iodoacetamide (IAA) to a final concentration of 40 mM, and incubation for 30 min in the dark at room temperature. To quench the reaction, DTT was added to a final concentration of 5 mM. Porcine trypsin (1:50, *w/w*) (Promega, Fitchburg, WI, USA) was added, and the mixture was incubated overnight at room temperature. The resulting peptide mixtures were desalted with hydrophilic–lipophilic-balanced solid phase extraction (Waters) and peptides eluted with 1 mL of 70% (*v/v*) acetonitrile and 1% (*v/v*) trifluoroacetic acid (TFA). Two biological replicates and two technical replicates were acquired for each condition.

### 2.3. Mass Spectrometry-Based Analysis of T. cruzi Tryptic Peptides

The peptides were separated by nano-liquid chromatography tandem mass spectrometry (nLC-MS/MS) on an EASY-Spray PepMap^®^ 50 cm × 75 μm C18 Column using an Easy nLC1000 nanoflow system (Thermo Fisher Scientific, Waltham, MA, USA). The high-performance liquid chromatography (HPLC) gradient was 5–25% solvent B (A 0.1% formic acid; B 100% acetonitrile (ACN), 0.1% formic acid) for 90 min at a flow of 300 nL/min. Mass spectrometric analysis was performed using an Orbitrap Fusion Tribrid mass spectrometer (Thermo Fisher Scientific); the maximum total cycle time was confined to 3 s. The most intense precursors selected from the FT MS1 full scan (resolution 120,000 full width at half-maximum (FWHM) @ *m*/*z* 200) were quadrupole-isolated and fragmented by collision-induced dissociation (CID) and detected in the dual-pressure linear ion trap with 30 as normalized collision energy. The MS1 scan range was between 380–1500 *m*/*z*, the ion count target was set to 2 × 10^5^, the MS2 ion count target was set to 1 × 10^4^, and the max injection time was 50 ms and 35 ms for MS1 and MS2, respectively. The dynamic exclusion duration was set to 10 s with a 10-ppm tolerance around the selected precursor and its isotopes. Two liquid chromatography tandem mass spectrometry (LC-MS/MS) experiments were performed for each biological replicates of the two life cycle stages of *T. cruzi*. The mass spectrometry proteomics data have been deposited to the ProteomeXchange Consortium via the PRIDE [22] partner repository with the dataset identifier PXD010768.

### 2.4. Protein Identification and Quantification

LC-MS/MS raw files were imported into MaxQuant version 1.5.2.8 [23] for identification and label-free quantification (LFQ) of proteins and peptides. MS/MS spectra were searched against the combined Uniprot *T. cruzi* CL Brener (downloaded, February 2018; 19,244 entries) and common contaminants protein database with a mass tolerance level of 4.5 ppm for MS and 0.5 Da for MS/MS. Enzyme specificity was set to trypsin, with a maximum of two missed cleavages. The carbamidomethylation of cysteine (57.0215 Da) was set as a fixed modification, and the oxidation of methionine (15.9949 Da), deamidation NQ (+0.9840 Da), and protein N-terminal acetylation (42.0105 Da) were selected as variable modifications. The minimum peptide length was set to seven amino acid residues. The match between runs feature of MaxQuant, which enables peptide identifications between samples based on their accurate mass and retention time, was applied with a match time window of 0.7 min and an alignment time window of 20 min. All of the identifications were filtered in order to achieve a protein false discovery rate (FDR) of 1% [24,25,26].

Proteins identified in the reverse database, and contaminants identified only by site were removed before performing statistical analysis. The dependent peptide algorithm was activated.

Proteins were quantified based on non-modified and N-terminally acetylated peptides. Oxidized and deamidated peptides were not considered for protein quantification, since these could have been raised from chemical artefacts, affecting the accuracy of protein quantification. Proteins quantified in two replicates of at least one biological condition were considered for further analyses. Proteins that appeared in two biological replicates of one condition and were not identified in the other biological replicate were considered exclusive proteins.

### 2.5. Bioinformatic Analysis

Statistical analyses of the proteome data were performed using Perseus v.1.5.1.6 [27]. To determine proteins with significant changes in abundances, *t*-test analysis was applied with permutation-based FDR correction at a FDR of 5%. Functional enrichment analysis was performed using Protein Center software (Thermo Fisher). The mRNA levels of some proteins discussed in this manuscript were retrieved from the TriTrypDB [28,29,30].

To evaluate the domains represented in the regulated uncharacterized proteins in the exponential and stationary growth phases, a standalone RPS-BLAST (v2.2.31+) was performed using the uncharacterized proteins in FASTA format as query, a dataset of position-specific scoring matrix (PSSM) [31] from CDD (Conserved Domain Database) [32] as database and 1 × 10^−5^ as e-value threshold.

Sequence homology between human and *T. cruzi* *N*-α-acetyltransferases was analyzed using BLASTP against *T. cruzi* strain CL Brener (taxid:353153) from the non-redundant database in NCBI [33].

### 2.6. Sequence Search and Phylogenetic Analysis

Two proteins detected in our MS/MS quantification analysis (a putative Nat1; accession number Q4D4R3 (Tc00.1047053504163.110) and an uncharacterized protein with the WD40 domain; accession number Q4DEL9 (Tc00.1047053507941.60) were used to perform phylogenetic analysis on *T. cruzi* genetic subdivisions termed discrete typing units (DTUs), *T. cruzi marinkellei*, *T. dionisii*, *T. erneyi*, and *T. rangeli*. Orthologs of selected proteins were obtained in the available genomes (completed and drafts) using a standalone tBLASTn (v2.2.31+). The genome from *T. cruzi* (JR cl. 4), *T. cruzi* (Sylvio X10/1), *T. cruzi* (CL Brener), *T. cruzi marinkellei* (B7), *T. cruzi* (Esmeraldo), and *T. rangeli* (SC58) are available in TriTrypDB (http://tritrypdb.org/tritrypdb/). *T. cruzi* (Tula cl2), *T. cruzi* (Bug2148), *T. cruzi* (Y), and *T. cruzi* (231) genome drafts are available in NCBI-Genbank [33]. The genome drafts from *T. dionisii* (TCC211), *T. erneyi* (TCC1946), *T. rangeli* (AM80), *T. cruzi marinkellei* (TCC344), *T. cruzi* (G)(TCC874), and *T. cruzi* (M-6241 TCC 2123) have been generated in our laboratories within the assembling the tree of life (ATOL, NSF-USA) and Trypanosomatid Culture Collection of the University of São Paulo (TCC-USP) (Brazil) projects, and were obtained as previously described [34].

Obtained sequences were aligned using MUSCLE v3.8.31 [35] and visualized using Seaview v.4.5.4 [36]. Inferred phylogenies were obtained by the maximum likelihood (ML) and parsimony (P) methods using Randomized accelerated Maximum Likelihood (RAxML) v8.2.4-1 [37] and Tree Analysis Using New Technology (TNT) v1.1 [38]. To infer ML phylogeny, the GTRGAMMA model was used. Branch statistical supports were obtained using Bootstrap with 1000 replicates as implemented in RAxML and TNT. Obtained phylogenetic trees were visualized using Figtree v1.4.3 [39].

### 2.7. TcPRODH and TcMDH Enzymatic Activities Assays

**Figure 2:**
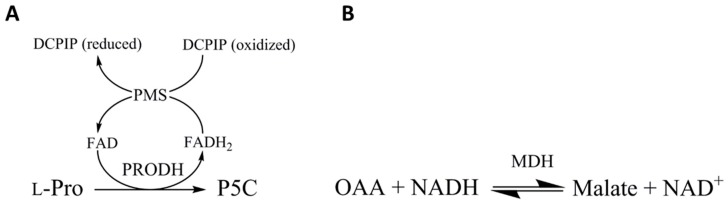
Enzymatic activities of proline dehydrogenase (PRODH) and malate dehydrogenase (MDH). The enzymatic activities were monitored by spectrometry by measuring the absorbance (OD) at 600 nm and 340 nm, respectively.

The enzymatic activities for both *T. cruzi* Proline Dehydrogenase (TcPRODH) and *T. cruzi* Malate Dehydrogenase (TcMDH) were measured in cell-free homogenates from epimastigotes in exponential and stationary phases (Figure 2). PRODH enzyme activity was measured by reducing a dye used as an electron acceptor, dichlorophenolindophenol (DCICP) (Figure 2A) [40]. The reaction contained 11 mM of 3-(*N*-morpholino) propanesulfonic acid (MOPS), 11 mM of MgCl_2_, 11% (*v/v*) glycerol, 0.26 mM of phenazin methosulfate (PMS), and 56 μM of DCICP; pH 7.5 and 60 µm of l-proline was initiated by the addition of the enzyme extract and the activity measured at optical density (OD) of 600 nm. malate dehydrogenase (MDH) enzyme activity was measured by oxaloacetate (OAA) reduction (Figure 2B). The reaction contained 100 mM of phosphate buffer, pH 7.5, 0.35 mM of OAA, and 0.12 mM of NADH, and was initiated by the addition of the enzyme extract [41]. The activity was monitored by measuring the absorbance (OD) at 340 nm.

### 2.8. Statistical Analysis

An unpaired *t*-student test was used to analyze the differences between groups using Perseus v.1.5.1.6 [27]. *p*-value < 0.05 was considered statistically significant.

## 3. Results and Discussion

### 3.1. Morphological and Molecular Characterization of T. cruzi in the Exponential and Stationary Phases

This study aimed at dissecting the molecular pathways activated in *T. cruzi* epimastigotes upon transitioning from the exponential to the stationary phase. The morphology of *T. cruzi* epimastigotes in the exponential and stationary phases present important differences. During the stationary phase, the parasite shows a slender shape with an elongated cellular body and flagellum (Figure 1C). A longer flagellum has been associated with limited glucose availability, which is a condition present during the stationary phase [42]. Moreover, a longer flagellum increases the ability to adhere to hydrophobic substrates and initiate the metacyclogenesis [43]. An increase in nucleotides, amino acids, and sugar transporters could facilitate the parasite survival during the stationary phase. The nucleolus of epimastigotes in the exponential phase are large, while in the stationary phase, they are reduced with the nucleus dispersed [44,45]. These morphological changes are exacerbated in the metacyclic trypomastigote, and have been associated with a reduced ribosomal protein content [46].

The following paragraphs will elucidate the protein-based molecular features of the *T. cruzi* in the exponential and stationary phases.

### 3.2. Differential Protein Expression in the T. cruzi Exponential and Stationary Phases

In this study, we applied quantitative mass spectrometry-based proteomic analysis to identify and quantify proteins present in the exponential and stationary phase of *T. cruzi* (Figure 1A). *T. cruzi* cells were collected during the exponential (from two to four days) and stationary phase (from five to eight days) (Figure 1B); then, they were lysed, and the extracted proteins were digested with trypsin. Tryptic peptides were analyzed by large-scale mass spectrometry-based proteomics and bioinformatics, and functional analyses were performed (Figure 1A).

**Figure 3:**
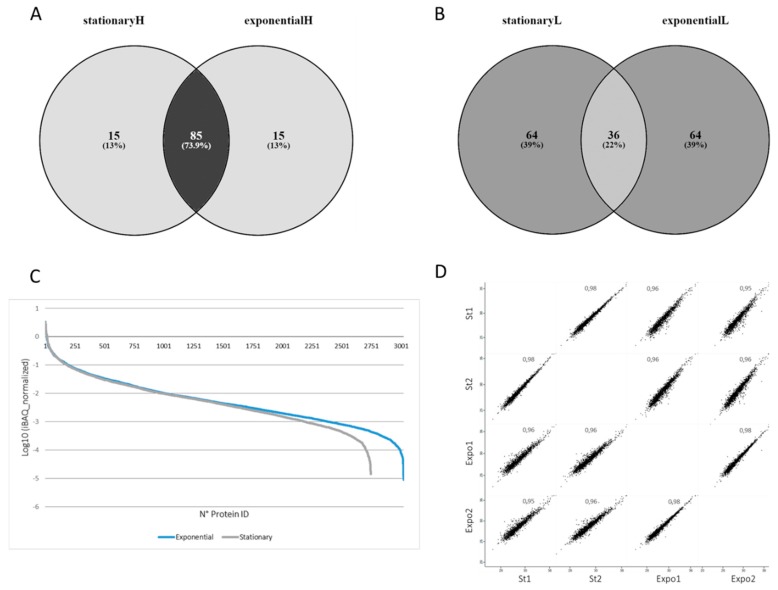
Proteome coverage and expression in the *T. cruzi* exponential and stationary phases. (**A**) intensity-based absolute quantification (iBAQ) values for the top 100 most abundant and (**B**) lowest abundant proteins identified in the stationary and exponential phases, H and L, respectively. iBAQ values were derived from LC-MS/MS values using the MaxQuant computational platform. (**C**) Normalized iBAQ values for the proteins identified and quantified in the exponential and stationary phases. Normalized iBAQ values were obtained by dividing each value for the total iBAQ in each biological replicate and averaging the two values. (**D**) Pearson correlation profile between the quantified proteins identified in the two biological replicates of the exponential (Expo1 and Expo2) and stationary phases (St1 and St2).

A total of 3440 proteins were identified. Of these, 3421 proteins were identified with at least one unique peptide, and 2720 proteins were identified with more than two peptides (Table S1). In particular, 1675 and 1721 proteins were identified in the biological replicate 1 and 2 of the stationary phase, and 2117 and 2509 proteins were identified in the biological replicate 1 and 2 of the exponential growth phase (Table S1), respectively. Of the total proteins identified, 1712 proteins were annotated as uncharacterized, corresponding to 50% of the total protein identified. These results confirm the percentage of hypothetical and uncharacterized proteins annotated in the *T. cruzi* proteome [46]. A total of 14,846 peptides were identified with an average of four peptides per protein. These results indicate a high proteome coverage with a larger proportion of peptides identified in the exponential phase. The identified proteins spanned a diverse molecular weight range from 8 kDa (Ubiquitin, putative, Tc00.1047053447255.4) to 530 kDa (Uncharacterized protein, Tc00.1047053507083.109) (Table S1). A total of 3091 proteins were quantified with a mean standard error of the mean of 10% for the stationary phase and 12% for the exponential phase. The intrasample protein abundance was calculated using the intensity-based absolute quantification (iBAQ) value [47]. In particular, normalized iBAQ values were calculated for each protein in each biological replicate, and an average normalized iBAQ was derived for each condition. The 100 proteins with the highest and the lowest iBAQ value were analyzed (Figure 3A,B). Interestingly, there was a high overlap (74%) between the 10 proteins with the highest abundance in the stationary and exponential phases, while low overlap (22%) was observed for the lowest abundant (Figure 3C). Between the highest abundant proteins, there were histones, heat shock proteins, tubulins, calpains, tryparedoxins, and metabolic enzymes. Proteins with the lowest abundance in the stationary phase were uncharacterized DNA and RNA-binding proteins.

**Figure 4:**
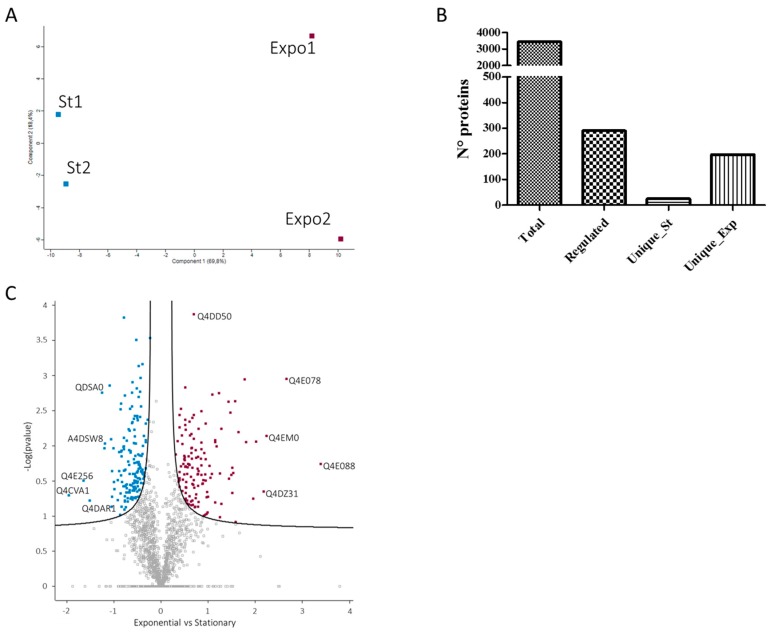
Quantitative analysis of proteins identified in the *T. cruzi* exponential and stationary phases. (**A**) Principal component analysis (PCA) analysis of label-free quantification (LFQ) values for the two biological replicates of exponential (Expo1 and Expo2) and stationary (St1 and St2) phases. (**B**) Number of total, regulated and unique proteins identified. (**C**) Volcano plot (Log2 (Exponential)-Log2 (Stationary) vs. –Log_10_ (*p*-value)) of quantified proteins in the exponential and stationary phases. Purple dots represent proteins upregulated in the exponential phase, while blue dots represent proteins upregulated in the stationary phase.

Relative protein abundance was assembled from the mass spectrometric signal of each corresponding peptide, identified, and matched across the different technical and biological replicates. High correlation was observed between the different biological replicates (0.98) and the different conditions (0.96) (Figure 3D). Principal component analysis (PCA) using 1459 proteins quantified in all of the biological replicates and in all of the conditions showed a clear separation between the two growth phases, indicating a complete remodeling of the *T. cruzi* proteome (Figure 4A). A total of 290 proteins were statistically regulated in the two conditions (FDR < 0.05), with 131 and 159 upregulated in the exponential and stationary growth phases, respectively (Figure 4C). Proteins upregulated in the exponential phase showed higher fold change compared to the ones upregulated in the stationary phase. Some of the highest upregulated proteins in the exponential phase were ribosomal proteins, while some of the highest upregulated proteins in the stationary phase were metabolic enzymes, indicating a stage-specific modulation of biological processes (Table S2). Moreover, 197 and 25 proteins were uniquely detected in all of the biological replicates in the exponential and stationary growth phases, respectively (Figure 4B). It should be noted that the identification in one condition does not imply the absence of that protein, but it is related to the abundance below the limit of detection and/or the presence of modified peptides (Table S3). This result is in apparent contrast with the observations of Parodi-Tolice et al., who found that almost 50% of the two-dimensional electrophoresis (2DE) spots were uniquely present in one stage [14]. However, our results are in agreement with the study from de Godoy et al. which detected a large proportion of proteins shared between the different metacyclogenesis stages [15]. This difference could be due to the technology used for detecting protein expression, 2DE versus shotgun, and more likely, to the presence of post-translational modifications that could be associated to stage-specific unique protein spots. Indeed, in a recent report, Amorim et al. have identified 260 phosphorylation events modulated during metacyclogenesis [18].

**Figure 5:**
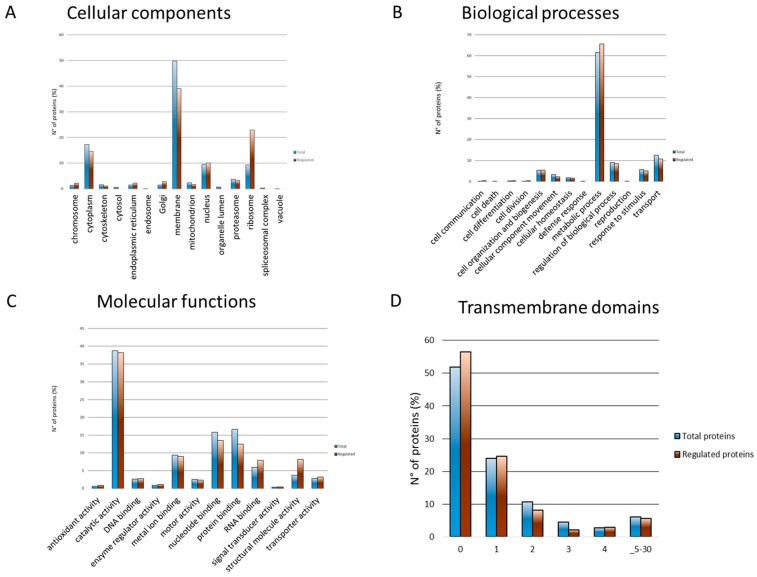
Gene ontology (GO) analysis and transmembrane domains of total and regulated proteins. (**A**) Cellular components, (**B**) biological processes, and (**C**) molecular functions were compared between the total and the regulated proteins. (**D**) Transmembrane domains were assigned to the total and regulated proteins. The gene ontology and transmembrane domain data were obtained using Protein Center software (Thermo Fisher).

The total and regulated proteins were mapped to gene ontology categories (Figure 5). The overrepresented categories were calculated based on the total *T. cruzi* CL Brener proteome (FDR ≤ 0.05). The regulated proteins presented higher enrichment in the ribosome, golgi, endoplasmatic reticulum, chromosome, and nucleus compared to the total proteins that were enriched more in the membrane and cytoplasm cellular compartments (Figure 5A). These data suggest the regulation of proteins involved in different subcellular compartments associated to specific biological functions. The regulated proteins were enriched in metabolic processes, cell division, cell differentiation, and communication compared to the total proteins, which were enriched more in cellular homeostasis, response to stimulus, and transport biological processes (Figure 5B). Moreover, the molecular functions enriched in the regulated proteins were the antioxidant, structural, and transporter activity along with DNA and RNA binding compared to the total proteins, highlighting the regulation of specific molecular functions during the transitioning from the two growth phases (Figure 5C). It should be noted that 50% of the proteins were classified as unannotated for their cellular components, biological processes, and molecular functions. This is in agreement with the large percentage of uncharacterized proteins identified. The unannotated proteins are discussed below.

**Figure 6:**
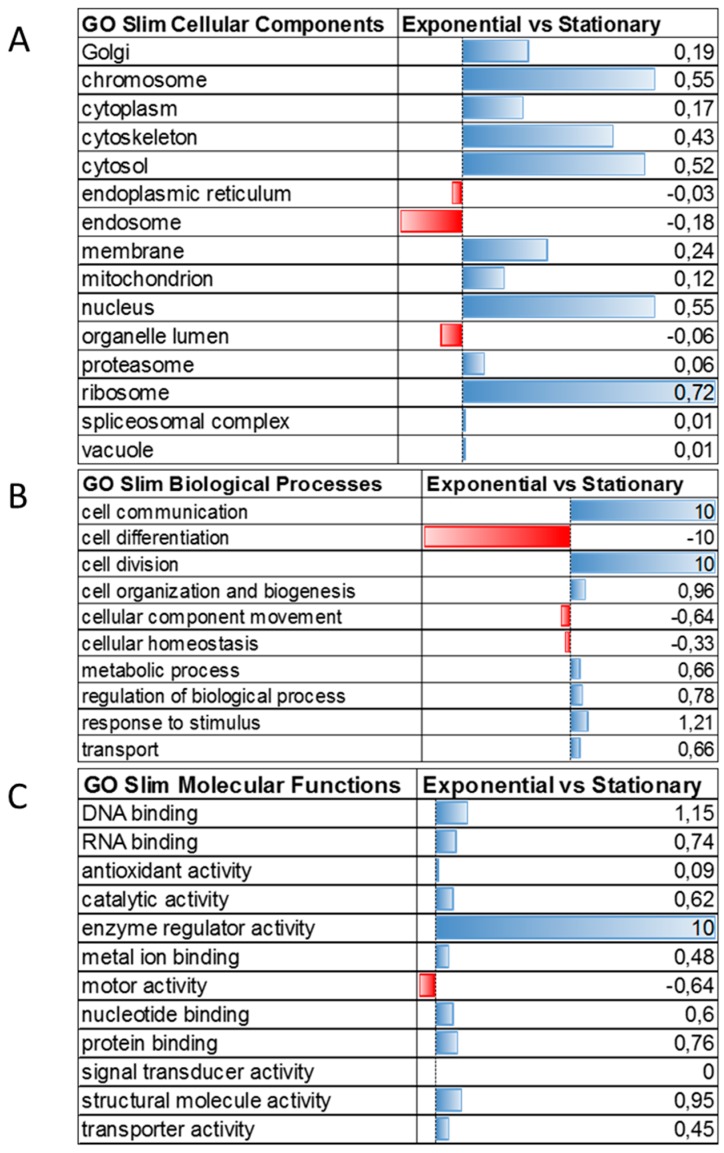
Gene ontology analysis combined with quantitative data of proteins differentially expressed between *T. cruzi* exponential and stationary phases. (**A**) Cellular components, (**B**) Biological processes and (**C**) Molecular functions of regulated proteins. Blue bars represent proteins upregulated in the exponential phase, while red bars represent proteins upregulated in the stationary phase.

Moreover, gene ontology categories were mapped against the protein expression values (Figure 6). Proteins associated to ribosome, nucleus, chromosome, cytosol, and cytoskeleton cellular components were upregulated in the exponential phase (Figure 6A). These data can be associated with the high expression of ribosomal proteins in the exponential phase. Indeed, 36 ribosomal proteins were upregulated in the exponential phase, and one was uniquely identified in the stationary phase (Table 1). Indeed, protein synthesis during growth phase transitioning is reduced as shown by the lower incorporation of [35S]-methionine in *T. cruzi* epimastigotes entering the logarithmic growth phase [48]. The downregulation of protein synthesis during *T. cruzi* transitioning from the exponential to the stationary phase supports the lower metabolic activity of the parasite during nutritional stress. On the contrary, proteins associated with the endoplasmic reticulum were upregulated in the stationary phase (Figure 6A, Table S2). Within the endoplasmic reticulum-associated proteins, calreticulin was found overexpressed twofold in the stationary phase compared to the exponential phase. Calreticulin is a ubiquitous calcium-binding chaperone involved in the correct folding of native glycoproteins. *T. cruzi* calreticulin (TcCRT) is a 45 kDa protein that binds monoglucosylated glycans of proteins to assure their proper folding, such as cruzipain [49]. Moreover, TcCRT is shuttled to the parasite surface interacting with the complement system. This interaction inhibits the complement system activation, facilitating the host immune system, and functioning as a molecular mimicry to invade the host cells increasing *T. cruzi* infectivity [50]. In our study, two calreticulin proteins (Q4DDX3; Tc00.1047053509011.40 and Q4CPZ0; Tc00.1047053510685.10) were upregulated in the stationary growth phase (Table S2). The upregulation of this protein could be related to its protective role against misfolded proteins and/or its role in infectivity. More experiments are needed to determine the localization and role of TcCRT during metacyclogenesis.

The biological processes upregulated in the exponential growth phase were cell division, cell communication, cell organization, and biogenesis. On the contrary, cell differentiation and cellular movement were upregulated in the stationary growth phase (Figure 6B). These results support the phenotypical evidence of replicative exponential form and differentiating stationary form. Enzyme regulator activity, DNA and RNA binding were upregulated in the proliferative phase, while the molecular process upregulated in the quiescent phase was motor activity (Figure 6C).

### 3.3. Membrane Proteins Regulation during Growth Phase Transitioning

Half of the total and regulated proteins identified in this study contained more than one transmembrane domain, indicating a remodeling of the membrane proteome (Figure 5D).

**Figure 7:**
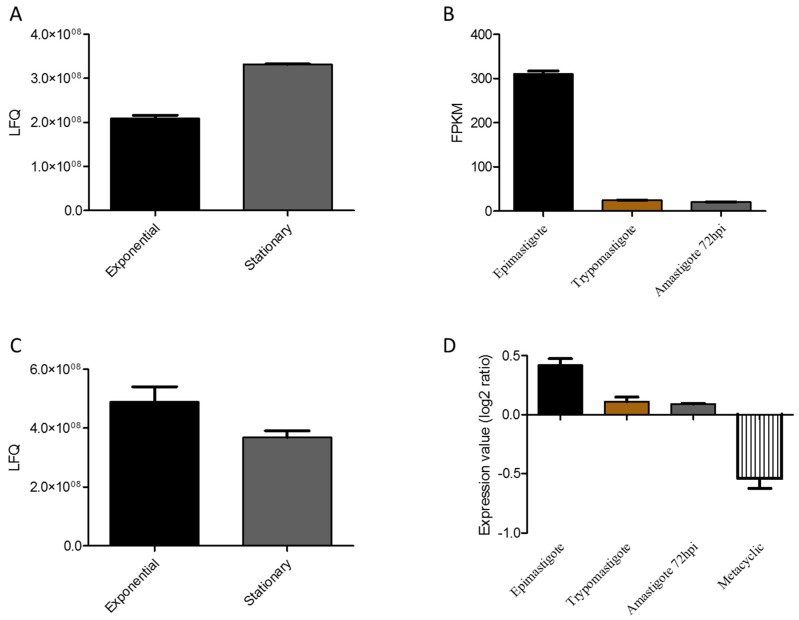
Protein and mRNA regulation of amastin and ADP/ATP mitochondrial carrier. (**A**,**C**) show the LFQ values for the amastin and ADP/ATP mitochondrial carrier protein in the *T. cruzi* exponential and stationary growth phases, respectively. These values were obtained by quantitative proteomics analysis in this study. (**B**,**D**) show the mRNA expression of amastin and ADP/ATP mitochondrial carrier in the epimastigote, trypomastigote, amastigote 72 h post infection (hpi), and metacyclic trypomastigote. These values were obtained from the TrytripDB database according to Mining et al. [28] and Li et al. [29].

One of the membrane proteins regulated during transitioning from the exponential to the stationary growth phase was amastin (Q4CVL1, Tc00.1047053509051.20), which was upregulated in the stationary phase (Supplementary Table S2, Figure 7). The amastin gene family includes 20 kDa surface membrane glycoproteins with a signal peptide, and four transmembrane domains with structural similarity to claudin [51]. This gene family is present in several trypanosomatids such as *Leishmania*, where α-, β-, γ-, and δ-amastin subfamilies are found [52], while in *T. cruzi*, the amastin gene family contains the two genes for the β- and four genes for the δ-amastin subfamilies. Amastin genes are highly expressed in the intracellular life stages of *T. cruzi* and *Leishmania* [53,54]. Interestingly, mRNA expression of different amastin subfamilies in CL Brener, Y, G, and Sylvio X-10 strains showed an upregulation in the amastigote form for the δ-amastins, while the β subfamilies were upregulated in the epimastigote form [28]. In this study, we detected the upregulation of the amastin β1 subfamily (Tc00.1047053509051.20) in the stationary growth phase compared to the exponential phase. Beside this gene, another member of the amastin β1 subfamily (Tc00.1047053509965.390) and amastin β2 subfamily (Tc00.1047053511497.19) were identified in the total proteome. None of them fulfilled the requirements to be selected for statistical analysis; however, Tc00.1047053511497.19 showed the same trend as Tc00.1047053509051.20 with upregulation in the stationary growth phase (Figure 7A).

These results are in apparent contrast with the mRNA results that showed upregulation of β-amastins in the epimastigote form (Figure 7B). However, a proteomic analysis of *T. cruzi* during metacyclogenesis showed the upregulation of members of β1-amastins (Tc00.1047053509051.20) in the metacyclic trypomastigotes compared to epimastigotes [15]. These results highlight a specific modulation of this gene family during metacyclogenesis and its potential involvement in parasite infectivity. In *L. braziliensis*, amastin knockdown impaired intracellular parasite growth in vitro, and did not cause infection in vivo [51]. These data point toward an important role of the amastin gene family in parasite survival, growth, and infectivity. More studies are needed to elucidate the functional role of amastins in *T. cruzi* biology.

Enzymes involved in the ATP metabolism were regulated during the exponential to stationary phase growth phase transitioning. The ADP/ATP mitochondrial carrier protein (AAC, uniprot ID: Q4DYK2, Tc00.1047053506211.160) was identified as upregulated in the exponential phase (Supplementary Table S2, Figure 7C). AAC belongs to the well-defined family of mitochondrial (mt) carrier proteins that are located in the inner mt membrane, and are involved in the transport of a wide range of metabolites [55]. This enzyme was found to be overexpressed in the procyclic form of *T. brucei* [56]. RNAi-mediated silencing of ADP/ATP carrier protein in the insect stage of *T. brucei* affected parasite growth, reduced the level of cytosolic ATP, and elevated reactive oxygen species [57]. The mRNA levels of the ADP/ATP mitochondrial carrier protein confirmed the proteomic data with downregulation in the metacyclic trypomastigote, amastigote, and bloodstream trypomastigote compared to the epimastigote stage (Figure 7D).

To evaluate the energy metabolism switch during the transition from exponential to stationary phases, the ATPase and ATP synthase enzyme LFQ values in the two phases were examined. In our analysis, three putative ATPases (TcCLB.506649.20/Q4DE37, TcCLB.509733.170/Q4DWB5 and TcCLB.509767.70/Q4DSC7) were upregulated in the stationary phase. Metacyclogenesis is characterized by the utilization of amino acids for the parasites’ cellular ATP provision. H+-ATPases catalyze the hydrolysis of ATP into ADP, a phosphate and a proton (H^+^), and the proton pumping across membranes which creates a proton motif force (energy) that is utilized by secondary transporters to transport ions and metabolites in and out of the cells [58]. On the other hand, mitochondrial membrane-bound ATP/ADP carrier protein 1 and a putative ATP synthase F1 alpha subunit were upregulated in the exponential growth phase. These enzymes are involved in mitochondrial proton transport. The downregulation of ATP synthases and upregulation of ATPases in the stationary phase (Table S2) are in accordance with the reduced intracellular ATP pool that is associated with starvation, reduced glycolysis, and motility in the quiescent state in T. cruzi [59].

Two gene products were associated to Gim5a and upregulated during the exponential to stationary phase transitioning. The first Gim5a protein (Q4E4Q2, Tc00.1047053508461.570) was upregulated in the stationary phase (Supplementary Table S2). This protein has a predicted molecular weight of 26 kDa and contains two transmembrane domains and no signal peptide. The other Gim5a gene product (Q4CR45, Tc00.1047053510669.49) was only found in the stationary phase. This protein has a predicted molecular weight of 17 kDa and has one transmembrane domain. Both proteins contain the Peroxisomal biogenesis factor 11 (PEX11) domain [60]. PEX proteins are involved in microbody biogenesis and the import of matrix proteins [61,62,63]. In *T. brucei*, the three most abundant glycosomal membrane proteins are PEX11 isoforms [64,65]. The *TbPEX11* gene encodes for a 24-kDa glycosomal membrane protein that is essential in trypanosomes. The modulation of this protein caused the remodeling of glycosomal size and number in *T. brucei*, being vital for its survival. The other two proteins are glycosomal integral membrane (GIM) proteins, GIM5A and GIM5B [66]. The depletion of GIM5B is lethal for the bloodstream form of *T. brucei*, while the procyclic form is able to survive in the same conditions, indicating a GIM5-dependency based on the life stage [67]. Moreover, Gim5 depletion leads to changes in the morphology of glycosomes and mitochondria and the different expression of ether-linked lipids [67]. Little is known about the function of the Gim5 protein in *T. cruzi*. Interestingly, no Gim5b protein has been annotated in the *T. cruzi* proteome, while one protein was annotated in the *T. evansi* and two in the *T. grayi*. The mRNA levels of the Gim5a protein show an upregulation in the amastigote form after 48 h of infection compared to the epimastigote form of *T. cruzi* Y strain [29]. However, its role in modulating metabolic pathways and affecting glycosomal and mitochondria morphology is still unknown. During transitioning from the exponential to the stationary phase, the overexpression of the GIM5a protein in *T. cruzi* could be involved in the glycosomal integrity and supply of metabolites and energy for parasite survival. More experiments are needed to elucidate the role of this protein in *T. cruzi*.

Despite the morphological changes that *T. cruzi* undergoes when transitioning from the exponential to the stationary growth phase, no differences have been detected in the expression levels of actin and tubulin [68]. Indeed, Cevallos et al., using a polyclonal serum raised against a 44 kDa recombinant *T. cruzi* actin protein, observed no changes in actin expression in the two growth phases, despite a lower level of actin mRNA that was more stable in the stationary phase compared to the exponential phase [69]. This study confirmed previous findings since actin, actin-like, actin-related proteins, and tubulins were not found to be differentially expressed during transitioning from the exponential to the stationary phase (Table S2). Interestingly, 2DE highlighted differentially regulated actin proteoforms in the epimastigote, amastigote, and trypomastigote forms, suggesting the regulation of these structural proteins based on post-translational modifications [68].

Furthermore, we mapped the domains in the regulated proteins using the protein families (Pfam) domain database (https://pfam.xfam.org/). A total of 560 domains were detected in 395 proteins. The majority of the domains were associated to a single protein. However, the RNA recognition domains (PF00076 and PF14259) were identified in 10 and nine regulated proteins, respectively, confirming the regulation of proteins involved in RNA metabolism during transitioning from the exponential to the stationary phase (Figure S1). Interestingly, the autophagy protein Apg6 (PF04111) domain was associated with eight regulated proteins. Proteins involved in the formation of autophagosomes play an important role in the protein degradation process induced by starvation [70]. This domain was mapped to several uncharacterized proteins, which are discussed below.

### 3.4. The Uncharacterized Proteome Modulated during Transitioning from Exponential to Stationary Growth Phase

To gain a deeper understanding of the differentially regulated uncharacterized proteins’ functions during transitioning from the exponential to the stationary growth phase, we investigated their domains using standalone RPS-BLAST search using the conserved protein domains present in CDD. The uncharacterized proteins that were regulated and did not have: (1) primary sequence similarity to any known protein, (2) any previously annotated domain and (3) a specific gene ontology category classification, were analyzed by structural homology search. A total of 167 unique domains were assigned to 119 regulated uncharacterized proteins (Table S4), while 86 uncharacterized proteins could not be assigned to any known domain from CDD.

The most abundant domain represented in the uncharacterized upregulated proteins in the stationary phase was the APG6 conserved protein domain structure (pfam0411) with 39 APG-related domains belonging to four proteins (Q4DQS9, Q4CVE1, Q4CT11, and Q4CT56) compared to one APG domain found in Q4DSZ0 upregulated in the exponential phase (Table S4). Of the 39 APG domains, 23 domains belonged to Q4CT56, which was uniquely identified in the stationary phase with two unique peptides. Autophagy is an eukaryotic cellular response that degrades and recycles cytosolic components, including aged organelles, aberrantly folded proteins, or protein aggregates through lysosomal degradation [6]. Two ubiquitin-like conjugation systems, Atg12 and Atg8, are essential for the formation of autophagosomes in mammalian and yeasts cells [71,72]. The Atg12 system forms a 350-kDa complex (Atg12-Atg5-Atg16) in the cytosol that forms a pre-autophagosomal transient membrane coat that drives the deformation of the sequestering membrane during vesicle formation [72]. In *T. cruzi*, the Atg8 conjugation system made up of Atg3, Atg4, Atg7, and Atg8 has been identified in the parasite’s genome [73]. Further, Alvarez et al. demonstrated that autophagy is active in all four stages of the parasite, and is upregulated in events of starvation and differentiation [73]. During metacyclogenesis induced by nutritional and thermal stress, it has been demonstrated that autophagy is upregulated, and that the induction of autophagy promotes the metacyclogenesis of *T. cruzi* [74]. Host cell autophagy also plays a role in the lysosomal-dependent *T. cruzi* entry into host cells, as demonstrated by the presence of host cell autophagic protein LC3 in the vacuole containing *T. cruzi* and the recruitment of autolysosomes to parasite entry sites [75]. Meanwhile, the parasites autophagy process was shown to be upregulated in amastigotes after 72 h, when the intracellular amastins are undergoing differentiation [75]. The upregulation of this domain in the epimastigote stationary phase suggest the initial stages of autophagy induction during transitioning of *T. cruzi*. This is in agreement with Vanrell et al. [74], who describe autophagy as a key process in the transitioning of *T. cruzi* epimastigotes to metacyclic trypomastigotes.

The most abundant domain among the upregulated uncharacterized proteins in the exponential phase detected in the two growth phases was the WD40 domain Gβ repeat (pfam00400) with 12 domain hits belonging to four proteins (Q4DKP1, Q4DCC3, Q4DEL9, and Q4DCC3) (Table S4). Proteins with the WD (tryptophan/aspartic acid) domain play diverse biological functions, including cell division, gene transcription, transmembrane signaling, mRNA modification, apoptosis, cytoskeleton assembly, and sulfur metabolism regulation [76,77]. In addition, proteins belonging to this domain show unusual functional diversity considering their conserved sequence motif and tertiary structures [77]. More studies are needed to define the function of this domain during *T. cruzi* growth phase transitioning.

WD repeat proteins arose during the divergence of eukaryotes from prokaryotes or during early eukaryote stages [77]. This is corroborated by the presence of this domain in trypanosomes belonging to *Euglenozoa*, which is one of the first eukaryotes to diverge. *T. cruzi* is genetically highly diverse, and is divided into six discrete typing units (DTUs) termed TcI-TcVI [78], which infect man, and Tcbat [79], which is restricted to bats. Phylogenetic inferences based on the uncharacterized protein expressing the WD domain, Q4DEL9, confirm the relationships between *Schizotrypanum* species, placing *T. cruzi* DTUs clustering forming a monophyletic group and bat restricted trypanosomes *T. dionisii* and *T. erneyi* positioned basally to the lineage formed by *T. cruzi marinkellei* and *T. cruzi* DTUs. *T. cruzi* isolates clustered forming three groups. Isolates from the same DTUs did not cluster to form a monophyletic group; this has been noted in previous studies based on other molecular markers such as SSU rRNA. The incongruence between the phylogenetic relationships among DTUs is explained by the high conservation of this gene, showing low polymorphism levels to access inter-DTU phylogenetic relationships. Moreover, TcII and TcIII ipolyphyletic clustering with TcV and TcVI is due the hybrid nature of the DTUs TcV and TcVI.

### 3.5. Protein N-Terminal Acetylation in T. cruzi Epimastigote during Transitioning from Exponential to Stationary Growth Phase

Protein acetylation is among the most common co-translational and/or post-translational modifications of proteins [80]. The incorporation of acetyl (acyl) groups (-COCH_3_) from the donor molecule acetyl-Coenzyme A (acCoA) to the α-amino group of protein N-terminal (termed *N*-α/N^α^ /N-t acetylation) or to the ε-amino group of lysine residues (termed *N*-epsilon/N^ε^- or K-ε acetylation) is catalyzed by *N*-terminal acetyltransferases (NATs) and lysine acetyltransferases (KATs), respectively. *N*-α acetylation plays regulatory and functional roles, including DNA recognition, protein stability, protein–protein interactions, and protection from proteolytic degradation by the action of aminopeptidases, subcellular targeting, and protein folding [81]. K-ε acetylation functions in the regulation of transcriptional activation, cell cycle progression, and DNA repair [82,83].

The N-terminal acetylation irreversibly adds an acetyl group to the *N*-α-amino group of a polypeptide. It usually happens during translation when the first peptide emerges from the ribosome tunnel with the acetylation of methionine. Alternatively, the second amino acid of the protein can be Nt-acetylated after methionine removal by methionine aminopeptidases. This is called the *N*-methionine excision mechanism, and the most common acetylated residues are Ser, Ala, Thr, Val, and Pro. These residues are likely acetylated by a set of NATs that perform Nt-acetylation at specific amino acid residues.

**Figure 8:**
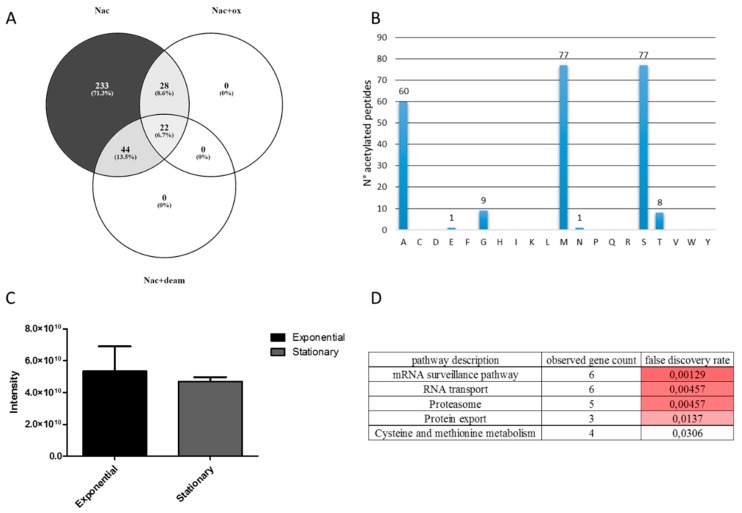
N-terminal acetylated peptides located at the protein N-terminal identified and quantified in the *T. cruzi* exponential to stationary phase transitioning. (**A**) Venn diagram of N-terminal acetylated peptides containing oxidation of methionine and deamidation of asparagine and glutamine. (**B**) Identity of the total N-terminally acetylated amino acids. (**C**) Intensity of only N-terminally acetylated peptides in the exponential and stationary phases. (**D**) Kyoto Encyclopedia of Genes and Genome (KEGG) pathways associated to N-terminally acetylated proteins.

A total of 327 unique N-terminal acetylated peptide sequences mapping to 306 proteins were detected. Within them, 66 were also N-terminally acetylated and deamidated in asparagine and glutamine residues, and 50 were N-terminally acetylated and oxidated in methionine residue (Figure 8A). Of these, 233 peptide sequences were only N-terminal acetylated, and considered for further analyses (Table S5). The amino acids that were identified to be N-terminally acetylated were methionine (77), serine (77), alanine (60), glycine (9), threonine (8), glutamic acid (1), and asparagine (1) (Figure 8B).

A gene ontology analysis of acetylated proteins revealed that the majority of the acetylated proteins were located mainly in the cytoplasm and intracellular organelles. The Kyoto Encyclopedia of Genes and Genome (KEGG) pathways associated with the N-terminally acetylated proteins were associated with RNA metabolism and proteasome (Figure 8D).

The total intensity of the acetylated peptides identified was higher in the exponential growth phase compared to the stationary, although this value was not significant (Figure 8C). Interestingly, 37 N-terminally acetylated peptides were detected only in the exponential phase, and two N-terminally acetylated peptides were detected only in the stationary phase, suggesting a high rate of protein N-terminal acetylation during the exponential phase (Table 2). Fourteen proteins were identified as N-terminally acetylated only in the exponential growth phase, while their protein abundance was similar in the two conditions leveraging the issue of the mass spectrometric limit of detection (Table 2). Within these proteins, we found acetyl-coenzyme A synthetase, adenylate kinase, and aspartate carbamoyltransferase. Moreover, aspartyl and phenylalanyl-tRNA synthetases were uniquely N-terminally acetylated in the exponential phase.

The adenylate kinase enzyme (Q4E1S6, Tc00.1047053506855.180) was found downregulated at the protein level in this study, and at the mRNA level in the exponential growth phase compared to the stationary phase [28]. However, the acetylated N-terminal residue of this protein was detected only in the exponential phase, suggesting a regulation of this protein through this co-/post-translational modification.

Three adenine phosphoribosyltransferases (Q4DVM9, Q4DNZ4, and Q4DVM8) were acetylated. The adenine phosphoribosyltransferase (Q4DNZ4; Tc00.1047053507519.140) was identified uniquely in the stationary phase, while the other two proteins were equally expressed in the two growth phases. Adenine phosphorybosyltransferase is involved in the purine salvage pathway, reutilizing purines and purine analogues from the host and synthesizing AMP from adenine [84,85,86]. This enzyme has been associated with benznidazol resistance in *T. cruzi*. Indeed, drug-resistant parasites have lower mRNA and protein expression of adenine phosphoribosyltransferase [87]. The expression of this enzyme and its regulation through acetylation might be an important drug target.

Two proteins were found to be N-terminally acetylated selectively in the stationary phase. In particular, the universal minicircle sequence binding protein (UMSBP) was detected acetylated uniquely in the stationary phase, while its protein expression was constant between the two growth phases. The universal minicircle sequence binding protein binds the universal minicircle sequence that constitutes the origin of the kinetoplast DNA replication [88]. Silencing of UMSBP in *T. brucei* inhibited minicircle DNA replication and parasite growth arrest [89]. The mRNA transcript expression of UMSBP in metacyclic trypomastigotes is drastically reduced compared to epimastigotes [28]. Moreover, a decrease in UMSBP expression was detected in *Leishmania donovani* after inhibiting spermidine synthesis through hypericin treatment [90]. In this study, we hypothesize that during the early signaling events of metacyclogenesis, the N-terminal acetylation of UMSBP could lead to protein degradation altering the replication of kinetoplastid DNA and arresting the parasite growth.

In higher eukaryotes, 6 *N*-acetyltransferase (NAT) complexes, NatA–NatF, have been described with different substrate specificity and subunit composition [91]. These enzymes catalyze the addition of an acetyl group to the primary amino group of the N-terminal protein residue using acetyl-CoA. We explored the sequence homology of the human *N*-alpha-acetyltransferases using BLASTP against *T. cruzi* NCBI non-redundant database. Human NatA (NAA10 (P41227), NAA11 (Q9BSU3), and NatB (NAA20 (P61599) sequences showed sequence identities of 54%, 57%, and 52% respectively to *N*-acetyltransferase complex ARD1 subunit from *T. cruzi* strain CL Brener. In yeast, ARD1 has been proposed to function by controlling transcription [92]. NAA15 (Q9BXJ9) and NAA16 (Q6N069), the human NatA auxiliary subunit proteins, had 28% protein sequence identity to *N*-acetyltransferase subunit Nat1 from *T. cruzi* strain CL Brener. ARD1 and NAT1 form the ARD1-NAT1 complex that is essential for *N*-terminal acetyltransferase enzyme activity [93]. Human *N*-α acetyltransferase NatC catalytic subunit ((NAA30/ Q147X3) had a 45% and 27% sequence identity to a *T. cruzi* hypothetical protein (Q4DGZ6) and a partial Nat complex ARD1 subunit (Q4D7K3), respectively. Sequence identity of the human NatE catalytic subunit NAA50 (Q9GZZ1) and NatF catalytic subunit NAA60 (Q9H7X0) to *T. cruzi* strain CL Brener shared a 35% and 29% sequence identity to an acetyltransferase (Q4DEI2) and an hypothetical protein (Q4CRN8/Q4DGZ6). No NAT-related hit was found between human NatD catalytic domain NAA40 (Q86UY6) and *T. cruzi.*

Initially, we screened the *T. cruzi* CL Brener genome, and found 18 sequences using the KEGG database. These sequences contain N-terminal acetyltransferase-related domains, as determined by the CDD database. Nine *N*-acetyltransferases were identified in our study (Table S6). In particular, the *N*-acetyltransferase subunit Nat1 was detected with 10 peptides that were unable to discriminate between the two gene products Tc00.1047053510301.80 and Tc00.1047053504163.110. The exponential/stationary ratio was 1.1, indicating no regulation during phase transitioning. The acetyltransferase putative (Tc00.1047053504867.40) was uniquely detected with two peptides and with an exponential/stationary ratio of 1.7. Although there was a trend in the upregulation in the exponential phase, this result was not statistically significant. Two RNA cytidine transferases (Tc00.1047053511303.90 and Tc00.1047053509177.30) were determined to contain the Nat domain, but no LFQ intensity was extracted. Two proteins were assigned as putative acetyltransferases (Tc00.1047053511311.80 and Tc00.1047053508831.120), but were not statistically regulated between the two conditions, leaving open the question about the regulation of these enzymes during transitioning from exponential to stationary phase.

Another possibility of lower protein N-terminal acetylation in the stationary phase might be related to the availability of acetyl-CoA. The expression of acetyl-CoA synthetase (Q4DQY7, Tc00.1047053509331.30) was 1.43 times higher in the exponential phase compared to the stationary (Table S2). A recent metabolomics analysis of *T. cruzi* epimastigotes in the exponential and stationary phase did not detect the intracellular levels of acetyl-CoA [11]. However, the levels of isoleucine and valine, branched amino acids involved in the biosynthesis of acetyl-CoA, were found downregulated in the stationary phase. Moreover, the level of pyruvate was found to be lower in the stationary phase compared to the exponential, supporting the hypothesis of a lower level of intracellular acetyl-CoA in the stationary phase. Cai L. and Tu B.P. measured the levels of acetyl-CoA in yeast cells cycling from growth and quiescent phases. They found that acetyl-CoA levels decrease in the stationary phase, leading to histones deacetylation and the upregulation of several genes related to starvation and survival [4,5], suggesting an explanation for the high levels of N-terminal acetylated proteins in the *T. cruzi* exponential phase. 

To our knowledge, this is the first study that investigated the N-terminal protein acetylation in *T. cruzi* during transitioning from exponential to stationary growth phases. Recently, Moretti et al. compared the N-terminal and lysine acetylation of *T. cruzi* and *T. brucei*. Here, 64 proteins were identified to be N-terminally acetylated in the epimastigote form of *T. cruzi* CL Brener with the majority moieties located in methionine, serine, and alanine [94]. The N-terminally acetylated proteins were associated to a diverse set of biological processes and compartments as shown in this study. It should be noted that in both studies, N-terminally acetylated peptides were not enriched. More analysis is needed in order to explore the N-terminal acetylome and clarify the enzymes involved in this process, their substrate specificities, and the biological effects of this modification in protists.

Phylogenetic analysis of one of the Nat1 genes quantified in our analysis, *Nat1* gene (Q4D4R3/Tc00.1047053504163.110), was performed on *T. cruzi* strains and closely related trypanosome strains. Our phylogenetic analysis based on the *Nat1* gene showed that the separation of *T. cruzi* DTUs from the closely related bat restricted T. c marinkellei, T. dionisii, and T. erneyi. The generalist T. rangeli was used as the outgroup of Schizotrypanum lineage (Figure S3). In addition, parental genotypes (TcII and TcIII) formed polyphyletic clustering with the hybrid DTUs (TcV and TcVI). However, this gene could not resolve between TCI, TCII, and TCV, showing TCI to be more closely related to TcV and TcII than to Tcbat. This indicates a non-conformity from the most common and well supported *T. cruzi* phylogenies [95]. The high sequence conservation of the *Nat1* gene among the *T. cruzi* DTUs could explain the incongruence between the phylogenetic relationships among the DTUs, with low polymorphism levels to access inter-DTU phylogenetic relationships. *T. cruzi* DTU clustering was congruent for both ML and P using RAxML and TNT (data not shown).

### 3.6. Methionine Oxidation in T. cruzi during Transitioning from Exponential to Stationary Growth Phase

The redox status of *T. cruzi* cells has been identified as a molecular switch to guide the proliferative to differentiation status of the parasite inside the triatomine vector. Indeed, Nogueira et al. suggested that *T. cruzi* epimastigote cells require an oxidative environment to proliferate while the differentiation process occurs in more reductive conditions [96]. The level of oxidized proteins in a biological system is strictly regulated by a balance of reactive oxygen and nitrogen species concentration, the antioxidant molecules concentration, and the activity of enzymes involved in the redox metabolism.

Initially, we focused on enzymes involved in the antioxidant molecules biosynthesis. Within the highest abundant proteins that were uniquely found in the exponential phase using the iBAQ value, there was cysthathionine beta-synthase (Q4DG21, Tc00.1047053508241.140) (Supplementary Table S1). Cystathionine beta-synthase (CBS) is a key enzyme of the transsulfuration pathway [97]. *T. cruzi* CBS mRNA is expressed in at least six independent isotypes with sequence microheterogeneity from tandemly linked multicopy genes, and it forms a homotetramer. The CBS enzyme has serine sulfhydrylase and cysteine synthase activities in vitro, and these activities are modulated in a stage-specific manner [29]. In particular, both activities were eight to nine times higher in the epimastigote than in the tissue culture-derived amastigote and trypomastigote. The mRNA level of CBS in *T. cruzi* Y strain during the infection of human fibroblasts was higher in the epimastigote stage than in the trypomastigote and amastigote, confirming the enzymatic activity results [29]. In our data, CBS was found to be 1.6 times more abundant in the exponential phase compared to the stationary phase, confirming the other observations (Table S2). These data could be explained based on the presence of low oxygen tension conditions during the stationary phase. Indeed, it was found that in yeast cells cultivated in aerobic conditions, CBS activity was three times higher [98]. Interestingly, cystathionine, a CBS product, was not regulated between the exponential and the stationary phase while homocysteine, serine, and cysteine—CBS substrates—were significantly upregulated in the stationary phase [11]. These results are apparently in disagreement between them; however, the lower expression of CBS in the stationary phase could be a way to keep the thiol-containing amino acids pool at higher concentrations in this growth phase. Moreover, we identified a 1.5-fold upregulation of trypanothione synthetase (Q9GT49) in the exponential phase (Table S2). Trypanothione is the main antioxidant molecule in the redox metabolism of trypanosomatids, and was found to be upregulated in the exponential phase [11]. Based on these data, it is not possible to deduce the redox balance of the two growth phases, since the cysteine and homocysteine are upregulated in the stationary phase, and the trypanothione is upregulated in the exponential phase.

In order to evaluate the oxidation state of the *T. cruzi* proteins during growth phase transitioning, we focused on the methionine-oxidized peptides. It should be noted that methionine oxidation is a process induced by sample preparation in a common bottom–up proteomic experiment [99]. However, differential methionine oxidation can be observed in samples subjected to oxidative stress. Due to that, we mapped the peptides oxidized in the methionine residues. First, 275 methionine oxidized peptides were detected, of which 271 had a localization probability of more than 0.75. From them, 134 methionine-oxidized peptides were present in the two biological replicates for at least one biological condition, and were considered for further analyses (Table S7). The total intensity of methionine-oxidized peptides showed a slight increase in the stationary phase compared to the exponential phase; however, this difference was not significant (Figure S4). The distribution of the Exp/St ratio of the methionine oxidized peptides showed a trend toward the stationary phase (Figure S4). Interestingly, two peptide methionine sulfoxide reductases (Tc00.1047053509611.180 and Tc00.1047053506945.160) were detected as significantly upregulated in the exponential phase.

More studies are needed to elucidate the oxidation state of different proteinogenic amino acids, such as the cysteine, tryptophan, and tyrosine side chains, during *T. cruzi* growth phase transitioning and their effect on the protein structure and function.

### 3.7. T. cruzi Malate Dehydrogenase and Proline Dehydrogenase are Regulated during Transitioning from Exponential to Stationary Phase

**Figure 9:**
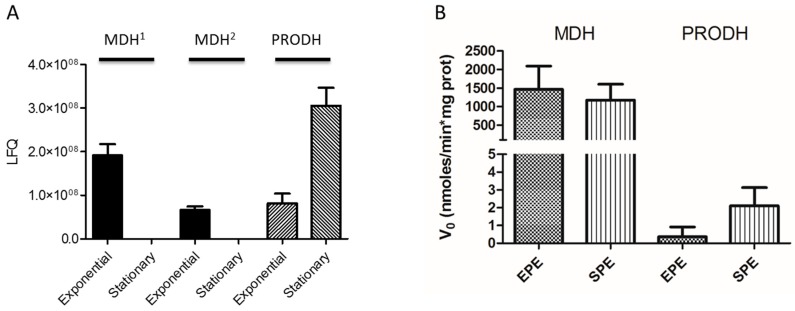
Malate dehydrogenase (MDH) and proline dehydrogenase (PRODH) expression and activity in the *T. cruzi* exponential and stationary phases. (**A**) LFQ expression of MDH and PRODH. MDH1 and MDH2 are two MDH isoforms associated to the Tc00.1047053506937.10 and Tc00.1047053507883.100 genes. MDH protein expression was only found in the exponential growth phase, while PRODH expression was upregulated in the stationary phase. (**B**) MDH and PRODH enzymatic activity measured in the exponential (EPE) and stationary (SPE) phases. The MDH was measured in the sense of the reduction of oxaloacetate (OAA) to form malate with the concomitant oxidation of NADH to NAD^+^. The consumed NADH was followed by spectrophotometric absorbance at 340 nm at constant temperature (37 °C). The PRODH enzyme activity was measured by the reduction of dichlorophenolindophenol (DCPIP) with the concomitant oxidation of L-Pro to 1D-Pyrroline-5-carboxylate (P5C). The reduction of DCPIP was monitored by spectrophotometric absorbance at 600 nm at constant temperature (37 °C). (**B**) The specific enzymatic activity of MDH and PRODH were measured in epimastigote cell-free extracts in exponential and stationary phase. No statistical difference between exponential and stationary phase were found for MDH and PRODH activities (MDH *p*-value = 0.5447 and PRODH *p*-value = 0.0612).

Exponential growth phase *T. cruzi* epimastigotes use different carbon and energy sources from those of the stationary growth phase. During the transition from exponential to stationary growth phase, the complete exhaustion of glucose leads to a metabolic switch from a metabolism based on glucose consumption to the usage of amino acids such as proline and histidine as energy sources [11,100]. During the exponential growth phase, glucose is converted into pyruvate by the glycolytic enzymes. Pyruvate can be fully oxidized in the mitochondrion through its conversion into acetyl-CoA, which fuels the TCA cycle. During this process, electrons from acetyl-CoA are transferred to oxidized nicotinamide adenine dinucleotide (NAD^+)^ and flavin adenine dinucleotide (FAD), forming the reduced NADH and FADH_2_, and are finally fed into the respiratory chain for oxidative phosphorylation (OXPHOS). As the first steps of the glycolysis are compartmentalized in the glycosome (a peroxysome-derived organelle), which is not able to exchange its pools of NAD^+^/NADH, the produced NADH must be re-oxidized inside the glycosome to keep the glycolysis working. As part of the reactions of the TCA cycle, the malate produced in the mitochondrion is oxidized to oxaloacetate by the mitochondrial MDH. Oxaloacetate can be converted into aspartic acid (Asp) to exit the mitochondrion through the malate/aspartate shuttle. Once in the cytoplasm, Asp can be a substrate of the aspartate or the tyrosine aminotransferase, which are able to transfer the –NH_2_ group from Asp to an acceptor ketoacid (pyruvate, to form alanine, or alpha –ketoglutarate, to form glutamate), releasing oxalacetate. Oxalacetate can freely diffuse into the glycosome, where it can be reduced to malate by the glycosomal MDH, with the concomitant reoxidation of NADH to NAD^+^. This complex cycle has both the mitochondrial and the glycosomal MDH as key players, and can constitute a mechanism regulating the intra-glycosomal redox state in order to maintain the glycolytic flux. As mentioned earlier, under glucose exhaustion, proline becomes one of the most relevant carbon and energy sources. Proline is well known as a “multi-functional metabolite” in *T. cruzi*. It is involved in several essential aspects of the parasite’s life cycle, such as the differentiation from epimastigotes to metacyclic trypomastigotes, and among intracellular stages, the host–cell invasion, and survival to thermal and nutritional stress as well as to oxidative imbalance [20,59,101,102,103]. The catabolism of Pro involves its oxidation into glutamate. This pathway is catalyzed by a FAD-dependent proline dehydrogenase (PRODH) (Q4CVA1; Tc00.1047053506411.30) and a NAD(P)+-dependent Δ1-Pyrroline-5-Carboxylate Dehydrogenase (P5CDH) [20,104]. Both steps are able to fuel electrons directly in the respiratory chain, which enables supporting ATP biosynthesis, even if the TCA cycle is not functional. However, proline can be fully oxidized: after being oxidized into glutamate, it can be deaminated to form the TCA intermediate alpha-ketoglutarate, which in turn can be converted into malate [100]. As mentioned above, malate can be a substrate of the malate dehydrogenase (MDH) to form oxaloacetate. Alternatively, if MDH is downregulated, it can be a substrate of the malic enzyme. In this case, it is converted into pyruvate and later acetyl-CoA, which can be fully oxidized in the TCA cycle. Noteworthy, if proline is being consumed instead of glucose, the need to reoxidate glycosomal NADH is predictably diminished, as it is the need of MDH activity. Summarizing, our work showed that two MDH isoforms (Q4CTR7 and Q4D4A1) were uniquely detected in the exponential growth phase, which is consistent with the predominance of glycolysis, while PRODH (Q4D4A1) is more expressed in the stationary phase, which is consistent with the predominance of proline catabolism (Figure 9A). Moreover, we measured the activity of these two enzymes and found a similar trend as reported for the protein expression (Figure 9B). The higher expression and activity of Δ1-Pyrroline-5-Carboxylate Dehydrogenase in the stationary phase was also confirmed by enzymatic activity assay, showing a regulated proline metabolism during transitioning from exponential to stationary phase (Figure 9, Figure S5) [11].

## 4. Conclusions

The fine regulation of metabolic and signaling pathways during growth phase transitioning of *T. cruzi* cells have been explored in this study. To our knowledge, this is the first study that interrogated the *T. cruzi* proteome upon transitioning from the exponential/proliferative to the stationary/differentiating phase in cell culture without externally induced stimuli. From more than 3000 proteins identified, 512 proteins were regulated at FDR <0.05. The findings reported in this study support the hypothesis that during the transition from the exponential to the stationary phase, *T. cruzi* activates a series of orchestrated multigenic/multiprotein events. Proteins involved in different metabolic pathways such as glycolysis and proline catabolism and the remodeling of cell surface and subcellular compartments are regulated during the two growth phases. These data complement the literature on morphological and metabolic changes during growth phase transitioning, offering a proteome-wide view on the key players in this process.

## Figures and Tables

**Table 1:** Ribosomal proteins regulated during transitioning from the exponential to the stationary growth phase. The exponential to stationary phase ratio is reported. Exp and St indicate proteins that were uniquely identified in the exponential and stationary phase, respectively.

Proteins	Name	Exp/St
**Q4E088**	40S ribosomal protein S10	10.09
**Q4E078**	Ribosomal protein L24	6.33
**Q4E3M0**	40S ribosomal protein S21	4.69
**Q4DZ31**	60S ribosomal protein L18a	4.75
**Q4D4L4**	40S ribosomal protein S11	3.53
**Q4DEK4**	40S ribosomal protein S16	2.87
**Q4D1T6**	Ubiquitin/ribosomal protein S27a	2.91
**Q4DJZ6**	Ribosomal protein L15 OS	2.78
**Q4DVM4**	60S ribosomal protein L7	2.43
**Q4DG45**	60S ribosomal protein L26	2.28
**Q4DVM5**	60S ribosomal protein L7	2.21
**Q4D5P4**	40S ribosomal protein S4	2.25
**Q4DI49**	60S ribosomal protein L23a	2.22
**Q4DTQ1**	40S ribosomal protein S23	2.02
**Q4DC23**	60S ribosomal protein L17	1.93
**Q4DPQ8**	60S ribosomal protein L2	1.93
**Q4CLU9**	40S ribosomal protein S8	1.91
**Q4CVQ4**	60S acidic ribosomal protein P2 beta (H6.4)	1.81
**Q4CQ63**	40S ribosomal protein SA	1.81
**Q4DW38**	40S ribosomal protein S24	1.74
**Q4E4R1**	60S ribosomal protein L23	1.73
**Q4DJY1**	Ribosomal protein S19	1.68
**Q4E3R2**	60S ribosomal protein L7a	1.64
**Q4DJX1**	Ribosomal protein L3	1.66
**Q4DZ41**	40S ribosomal protein S3a-2	1.62
**Q4DD50**	60S ribosomal protein L10	1.62
**Q4D6H7**	Ribosomal protein S20	1.56
**Q4E0N6**	40S ribosomal protein S15a	1.50
**Q4CTR5**	40S ribosomal protein L14	1.50
**Q4DGZ5**	40S ribosomal protein S15	1.47
**Q4DIZ9**	40S ribosomal protein S2	1.41
**Q4DIV9**	60S ribosomal protein L6	1.40
**Q4CU61**	60S ribosomal protein L5	1.39
**Q4D991**	60S acidic ribosomal protein	Exp
**Q4DTK4**	50S ribosomal protein L7Ae	Exp
**Q4DN72**	60S ribosomal protein L34	Exp
**Q4DMS9**	Ribosomal protein L27	Exp
**Q4CQG0**	40S ribosomal protein S12	St

**Table 2:** *T. cruzi* N-terminal acetylated peptides detected in one condition. Peptide sequences are reported. Nac_St and Nac_expo indicate the acetylated peptides in the stationary and exponential phase, respectively. Prot_St and Prot_expo indicate the average protein expression in the stationary and exponential phase. * Proteins quantified in two biological replicates in at least one condition were reported.

Peptide	Proteins	Name	Nac_St	Nac_exp	Prot_St *	Prot_expo *
AVVQTHCFNWMDHDGTR	Q4DMP7	Uncharacterized protein	0	340,000,000	1,100,000,000	1,480,000,000
MMEGFYGVEVASGQQVKPK	Q4D244	Nucleolar RNA-binding protein, putative	0	130,000,000	559,000,000	758,000,000
MENLTVEEAR	Q4DQY7	Acetyl-coenzyme A synthetase	0	107,000,000	1,710,000,000	2,450,000,000
ASVFYILDSK	Q4DX10	Mu-adaptin 1, putative	0	14,581,000	277,000,000	194,000,000
ATAAVALAPTADAAGSVLEPLLDK	Q4E342	Proteasome regulatory non-ATPase subunit 6, putative	0	56,185,000	340,000,000	599,000,000
STVEDFVLQALASTDAVESDK	Q4DY45	Phenylalanyl-tRNA synthetase alpha chain, putative	0	58,198,000	320,000,000	290,000,000
SDPDKSNTAASQEDASGNVASK	Q4CNC1	Aspartyl-tRNA synthetase, putative	0	58,968,500	745,000,000	688,000,000
SALTESLLDLHK	Q4E3B2	Uncharacterized protein	0	51,456,500	71,460,000	151,000,000
TAFLDNKLEYLQK	Q4E1S6	Adenylate kinase, putative	0	47,744,500	590,000,000	347,000,000
SLTLQSEQFQHIVR	Q4E093	40S ribomal protein S18, putative	0	60,961,500	3,230,000,000	3,550,000,000
SVFGVDFGNLNSTVAITR	Q4D673	Heat shock protein, putative (Fragment)	0	41,490,000	29,524,000	180,000,000
AALVHLPDPFVTLPFR	Q4D509	Uncharacterized protein	0	26,870,850	217,000,000	206,000,000
MLELPPVASLK	Q4D3W3	Aspartate carbamoyltransferase, putative	0	22,928,000	1,140,000,000	1,120,000,000
MIVLNGISEEQKK	Q4DDB3	Uncharacterized protein	0	32,419,000	598,000,000	408,000,000
TDKKEEQQNTEEYDYDR	Q4DTK4	50S ribomal protein L7Ae, putative	0	46,915,500	0	75,524,000
AEHLLEQLR	Q4D4P8	Uncharacterized protein	0	127,000,000	0	123,000,000
MDLDAFLNKK	Q4DUJ9	Uncharacterized protein	0	73,499,500	0	423,000,000
MFHGFPDVQIAPR	Q4E3S5	Uncharacterized protein	0	59,240,000	0	128,000,000
GDVEQIVEKEETDIQANVLAIPTFEAMGLK	Q4CMK2	ATP-dependent DEAD/H RNA helicase, putative (Fragment)	0	13,256,500	0	53,465,000
AELLTPK	Q4E1W2	Uncharacterized protein	0	13,818,000	0	70,917,500
MFFEGACAK	Q4CTR7	Malate dehydrogenase	0	26,409,500	0	193,000,000
MLNNELANLVDQQK	Q4DM75	Protein translation factor SUI1 homolog, putative	0	121,000,000		
SVAEGFLSHGEPCTR	Q4DV00	Uncharacterized protein	0	81,500,500		
MLHEIPLHDASASAAER	Q4CX95	Uncharacterized protein	0	74,033,000		
TAPSTTAASTAVPFLEVK	Q4CQ45	Glutaminyl-tRNA synthetase, putative (Fragment)	0	50,153,500		
SLEEVEPNFFTLSPDSPLR	Q4DVM8	Adenine phphoribyltransferase, putative	0	48,383,000		
AILPHGFIEAIHASPLR	Q4DS12	Uncharacterized protein	0	39,947,500		
METAPHNIQTER	Q4DS85	Uncharacterized protein	0	16,433,000		
SSDIIEHSFFFTPLER	Q4DZY5	Cholinephphate cytidylyltransferase A, putative	0	25,039,000		
SAFNPDAPAFIPTFLR	Q4DNJ8	RNA guanylyltransferase, putative	0	45,679,500		
MDFLHHVQGIVVLNELGGR	Q4DEQ7	Coatomer zeta subunit, putative	0	51,783,000		
MFDALSALVETASAK	Q4D4V0	Uncharacterized protein	0	40,160,500		
SGRPESVQGGNVESQSSNVSQGGR	Q4E0M5	Retrotranspon hot spot (RHS) protein, putative (Fragment)	0	50,721,500		
SHAGNVTNVYDEK	Q4DXE3	Uncharacterized protein	0	30,738,000		
AYNTPAWNEEFGVLK	Q4DJ63	Uncharacterized protein	0	28,215,500		
AALVHLPDQFVTLPFR	Q4DP85	Uncharacterized protein	0	33,409,000		
SDSTEQSVSKPETSK	Q4CMM3	Uncharacterized protein	0	28,615,500		
ADEVPMTKR	Q4D6T8	Universal minicircle sequence binding protein (UMSBP), putative	37,170,000	0	4,410,000,000	4,790,000,000
SLEEVEPNFFILSPDSPLR	Q4DNZ4	Adenine phphoribyltransferase, putative	117,000,000	0	186,000,000	0

## Supplementary Materials

The following are available online at http://www.mdpi.com/2073-4425/9/8/413/s1. Figure S1: PFAM domains associated to the number of proteins, Figure S2: Phylogenetic analysis of Q4DEL9 uncharacterized protein, Figure S3: Phylogenetic analysis of *T.cruzi* Nat1 (Q4D4R3), Figure S4: Expression of methionine oxidized peptides between the *T.cruzi* exponential and stationary phases, Figure S5: PRODH and MDH metabolic pathways regulated during *T.cruzi* transitioning from exponential to stationary phase, Table S1: Total proteins identified and quantified in the *T.cruzi* exponential and stationary phase, Table S2: Regulated proteins between the *T.cruzi* exponential and stationary phase, Table S3: Unique proteins identified and quantified in the *T.cruzi* exponential and stationary phase, Table S4: Regulated uncharacterized proteins between the *T.cruzi* exponential and stationary phase, Table S5: N-terminal acetylated peptides identified and quantified during transitioning from exponential to stationary phase, Table S6: Proteins with N-terminal acetyltransferase (Nat) domain, Table S7: Methionine oxidized peptides identified in the *T.cruzi* exponential and stationary growth phases.
