# Supplementary material for: Proteome-Wide Analysis of Trypanosoma cruzi Exponential and Stationary Growth Phases Reveals a Subcellular Compartment-Specific Regulation
Source: Genes (Basel). 2018 Aug 15;9(8):413. doi: 10.3390/genes9080413 (PMC6115888; doi:10.3390/genes9080413)

Supplementary information

Title: Proteome-wide analysis of *Trypanosoma cruzi* exponential and stationary growth phases reveals a subcellular compartment-specific regulation

Carla C. Avila[a°], Simon Ngao Mule[a°], Livia Rosa-Fernandes[a,b], Rosa Viner[c], Barison MJ[a], André G. Costa-Martins[a], Gilberto Santos de Oliveira[a], Marta M. G. Teixeira[a], Claudio R.F. Marinho[a], Ariel M. Silber[a] and Giuseppe Palmisano[a]*

[a] Department of Parasitology, Institute of Biomedical Sciences, University of Sao Paulo, Sao Paulo, Brazil

[b] Department of Biochemistry and Molecular Biology, University of Southern Denmark, Odense, Denmark

[c] Thermo Fisher Scientific, San Jose, USA

° These authors have contributed equally to the work.

*Correspondence to: Prof. Giuseppe Palmisano, Department of Parasitology, Institute of Biomedical Sciences, Avenida Lineu Prestes 1374, Butanta, University of Sao Paulo, Sao Paulo, Brazil

Supplementary information

Supplementary Tables

Supplementary Table 1: Total proteins identified and quantified in the *T. cruzi* exponential and stationary phase. Stationary phase 1 and 2 and exponential phase 1 and 2 indicates two biological replicates of each condition. Number of proteins, peptides, unique peptides, iBAQ, Intensity and LFQ values are reported.

Supplementary Table 2: Regulated proteins between the *T. cruzi* exponential and stationary phase. Stationary phase 1 and 2 and exponential phase 1 and 2 indicates two biological replicates of each condition. Number of proteins, peptides, unique peptides, Intensity and LFQ values are reported.

Supplementary Table 3: Unique proteins identified and quantified in the *T. cruzi* exponential and stationary phase. Stationary phase 1 and 2 and exponential phase 1 and 2 indicates two biological replicates of each condition. Number of proteins, peptides, unique peptides, iBAQ, Intensity and LFQ values are reported. The Log2 (Exponential-Stationary) values are reported as 30 when the protein was only identified in the exponential phase and -30 when it was only identified in the stationary phase.

Supplementary Table 4: Regulated uncharacterized proteins between the *T. cruzi* exponential and stationary phase. Uniprot accession number, domain description, expectation value, length and AA position are reported. The domains were retrieved by RPS-BLAST database containing the CDD, PFAM and Tiger domains. PFAM domain accession number are reported in the table.

Supplementary Table 5: N-terminal acetylated peptides identified and quantified during transitioning from exponential to stationary phase. Peptide sequence, protein accession number, protein description and peptide intensity are reported. Stationary phase 1 and 2 and exponential phase 1 and 2 indicates two biological replicates of each condition.

Supplementary Table 6: Proteins with N-terminal acetyltransferase (Nat) domain. The Nat genes were searched in KEGG database within the *T. cruzi* CL Brener strain sequences and each protein candidate was screened by the CDD domain database in NCBI.

Supplementary Table 7: Methionine oxidized peptides identified in the *T. cruzi* exponential and stationary growth phases. The methionine oxidized peptides identified with a PTM localization probability more than 0.75 are

reported in this table. The peptide intensity, sequence, position of oxidation within protein, uniprot accession number and protein description are reported. Stationary phase 1 and 2 and exponential phase 1 and 2 indicates two biological replicates of each condition.

Supplementary Figures

Supplementary Figure 1: PFAM domains associated to the number of proteins. The PFAM domains were retrieved using Protein Center software (Thermo Fisher) and were plotted against the number of proteins that contain them.

Supplementary Figure 2: Phylogenetic analysis of Q4DEL9 uncharacterized protein. The amino acid sequence of the Q4DEL9 was compared between different *T. cruzi* strains belonging to the seven DTUs and *T. c. marinkellei*, *T. dionisii*, *T. erneyi* and *T. rangeli* (outgroup). The sequences were retrieved from NCBI and aligned using MUSCLE algorithm and RAxML was used to infer maximum likelihood and build the phylogenetic tree.

Supplementary Figure 3: Phylogenetic analysis of *T. cruzi* Nat1 (Q4D4R3). The amino acid sequence of the Q4D4R3 was compared between different *T. cruzi* strains belonging to the seven DTUs and *T. c. marinkellei*, *T. dionisii*, *T. erneyi* and *T. rangeli* (outgroup). The sequences were retrieved from NCBI and aligned using MUSCLE algorithm and RAxML was used to infer maximum likelihood and build the phylogenetic tree.

Supplementary Figure 4: Expression of methionine oxidized peptides between the *T. cruzi* exponential and stationary phases. 134 methionine oxidized peptides with a localization probability more than 0.75 and identified in two biological replicates of at least one biological condition were quantified and compared between the two *T. cruzi* growth phases. A) Bar graph of intensity of 134 peptides detected in the two conditions. No significant difference was detected. B) Distribution of methionine oxidized peptides expressed as Log2 (Exponential/Stationary).

Supplementary Figure 5: PRODH and MDH metabolic pathways regulated during *T. cruzi* transitioning from exponential to stationary phase. In the presence of Glucose (Glc), as it happens during the exponential growth phase, Malate (Mal) can be obtained from the Pyr produced in the glycolisis, which is carried into the mitochondrion by a Mitochondrial Pyruvate Carrier (MPC), by feeding the Trycarboxilic acids (TCA) cycle with AcetylCoA (AcCoA). Mal can be converted into Oxaloacetate (OA) by the enzyme Malate Dehydrogenase (MDH), with the reduction of NAD+ to NADH. OA can be further oxidized in the TCA cycle, or aminated to form Aspartate (Asp), which can be exported from the mitochondrion into the cytoplasm through the Mal/Asp shuttle (MAS). Once in the cytoplasm, Asp can be deaminated back to OA by the Asp transaminase. The resulting OA can freely diffuse into the glycosomes, where glycosomal MDHs can reduce it into Mal, with the concomitant re-oxidation of NADH to NAD+, which is essential to maintain the glycolitic flux. In the absence of Glc, which happens during the late exponential and the starionary growth phases, L-Proline (L-Pro) is taken up from the extracellular medium or biosynthesized. The cytoplasmic L-Pro crosses the mitochondrial membranes and is converted into Δ1-Pyrroline-5-Carboxylate Dehydrogenase (P5C) by the enzyme Proline Dehydrogenase (PRODH) with the reduction of FAD to FADH2. P5C is further converted into L-Glutamate (L-Glu), with the reduction of NAD(P)+ to NAD(P)H. Both steps feed electrons into the respiratory chain. Glu is deaminated into the intermediate of the TCA cycle 2-oxoglutarate (2-OG). Through a sequence of reactions in the TCA cycle, occurs the formation of Mal which can be converted into Oxaloacetate (OA) by the enzyme MDH, with the reduction of NAD+ to NADH, or into Pyr by a malic enzyme, which can re-entry the TCA cycle though its conversion to AcCoA.

Supplementary Figure 1:

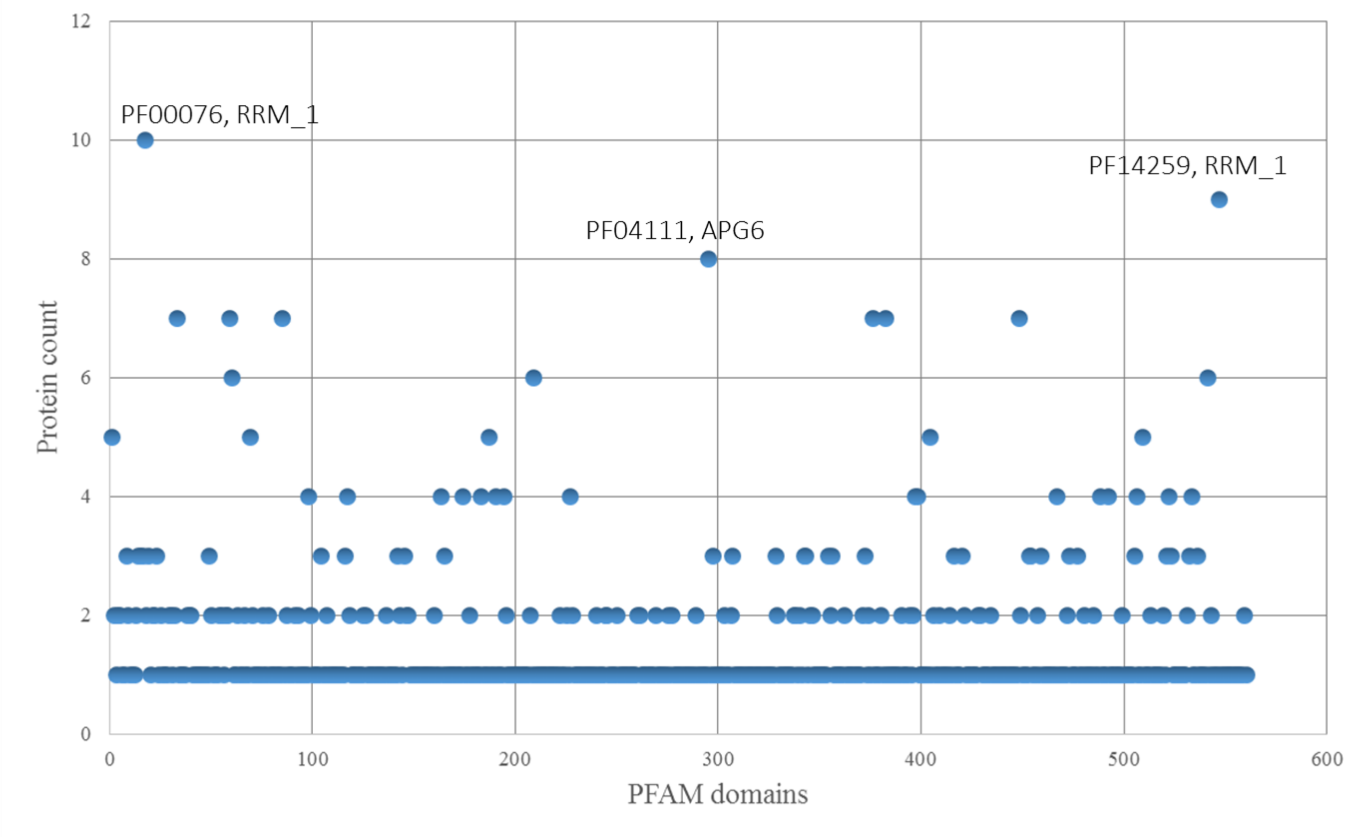

Supplementary Figure 2

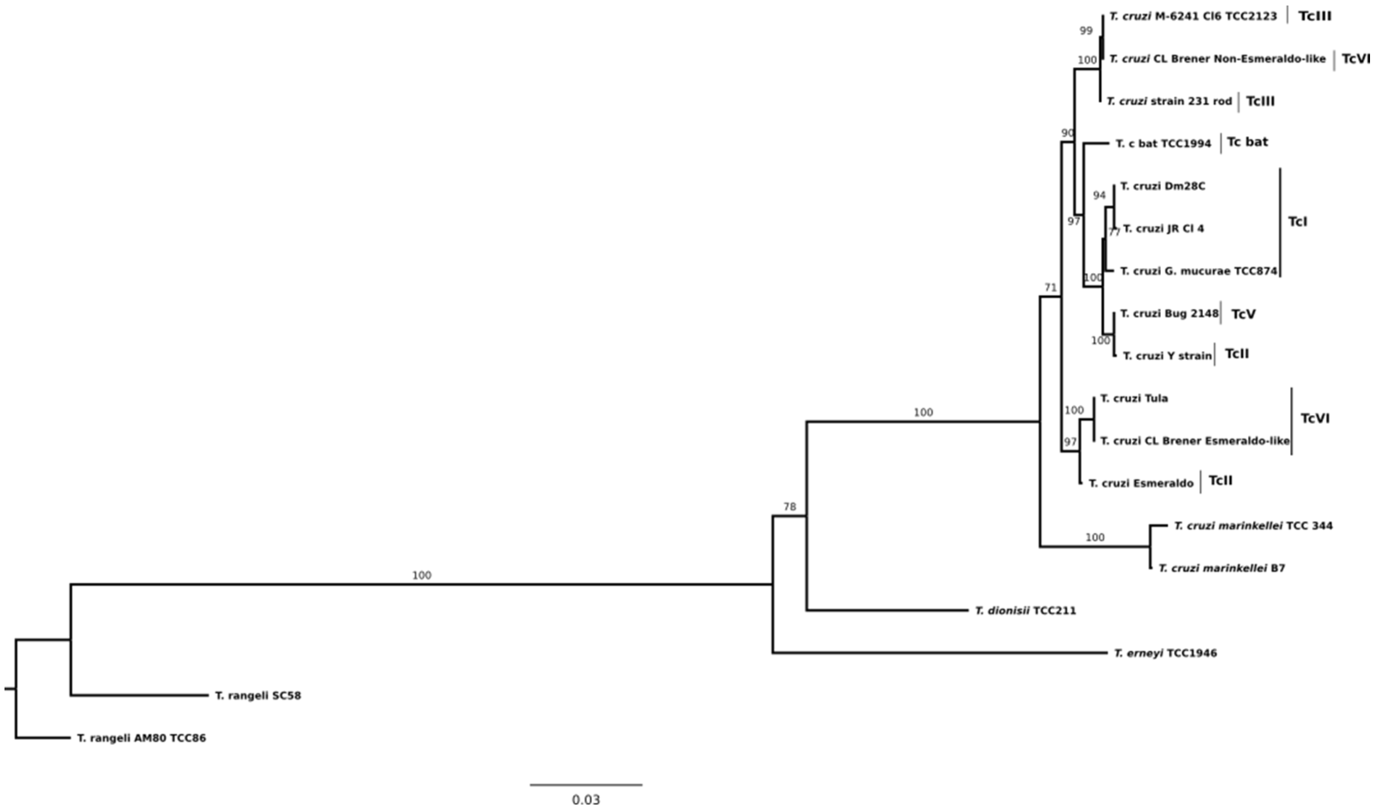

Supplementary Figure 3:

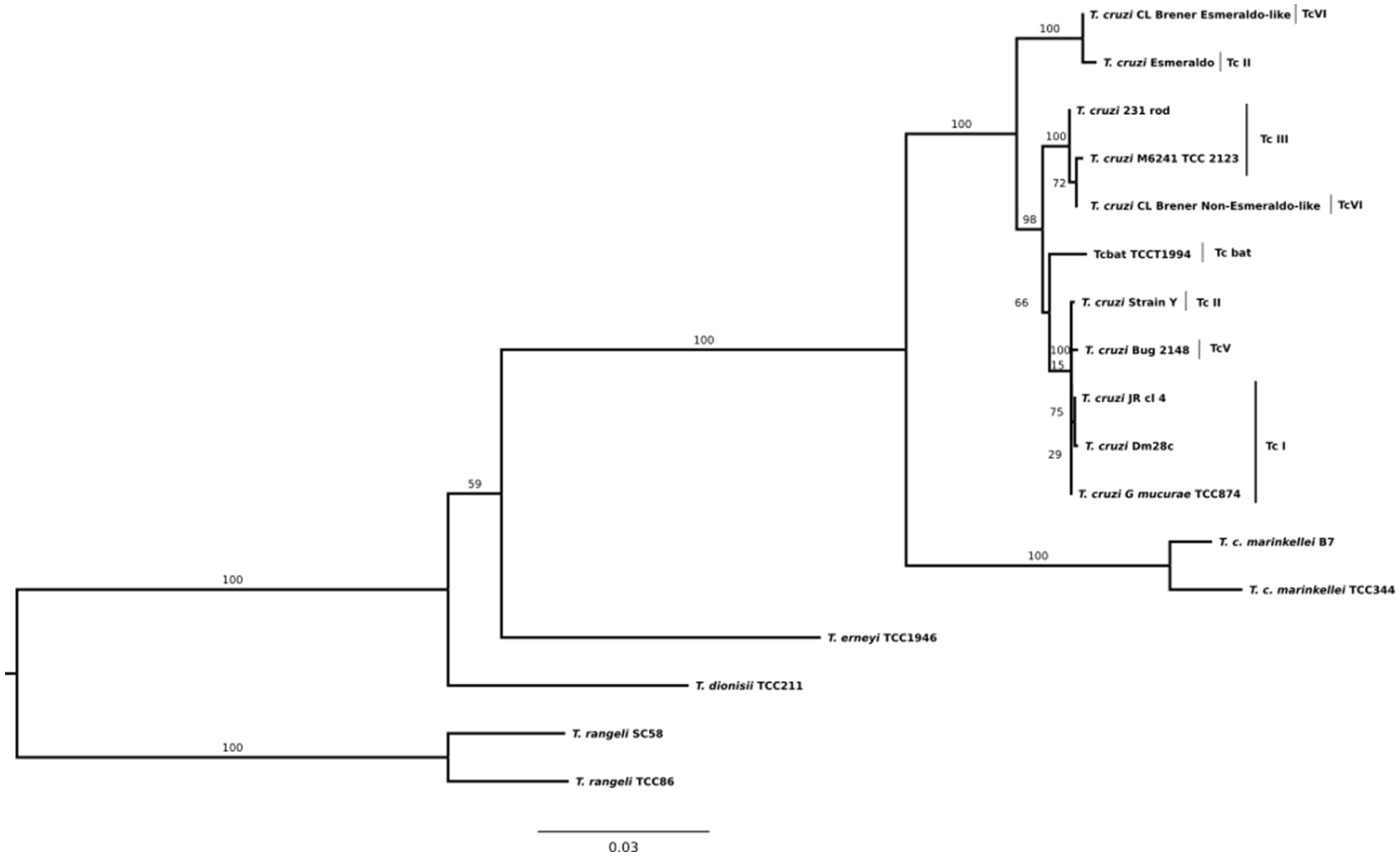

Supplementary Figure 4:

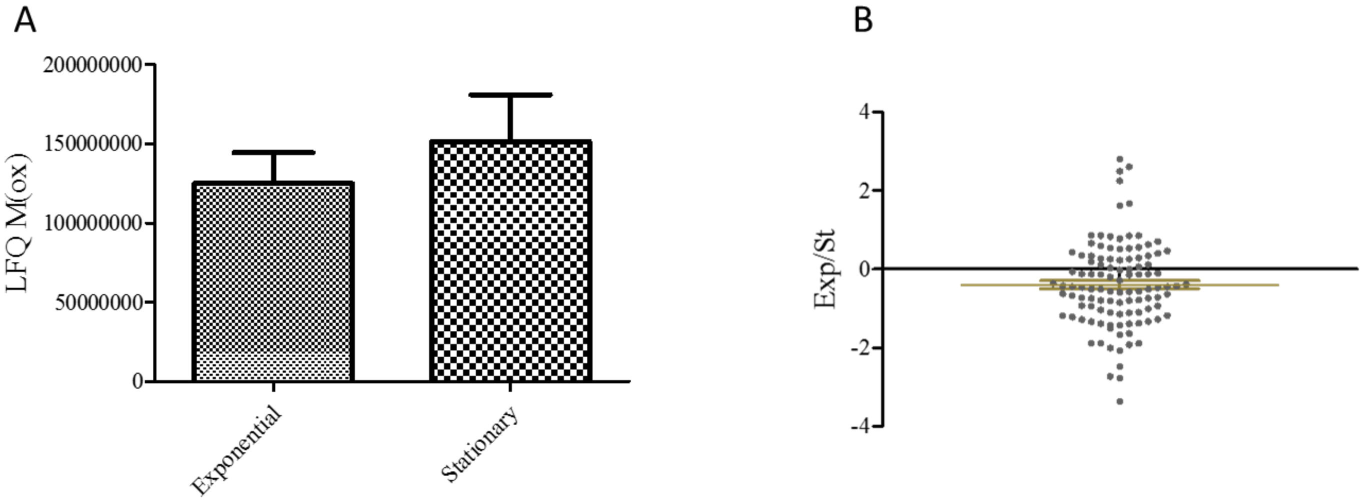

Supplementary Figure 5:

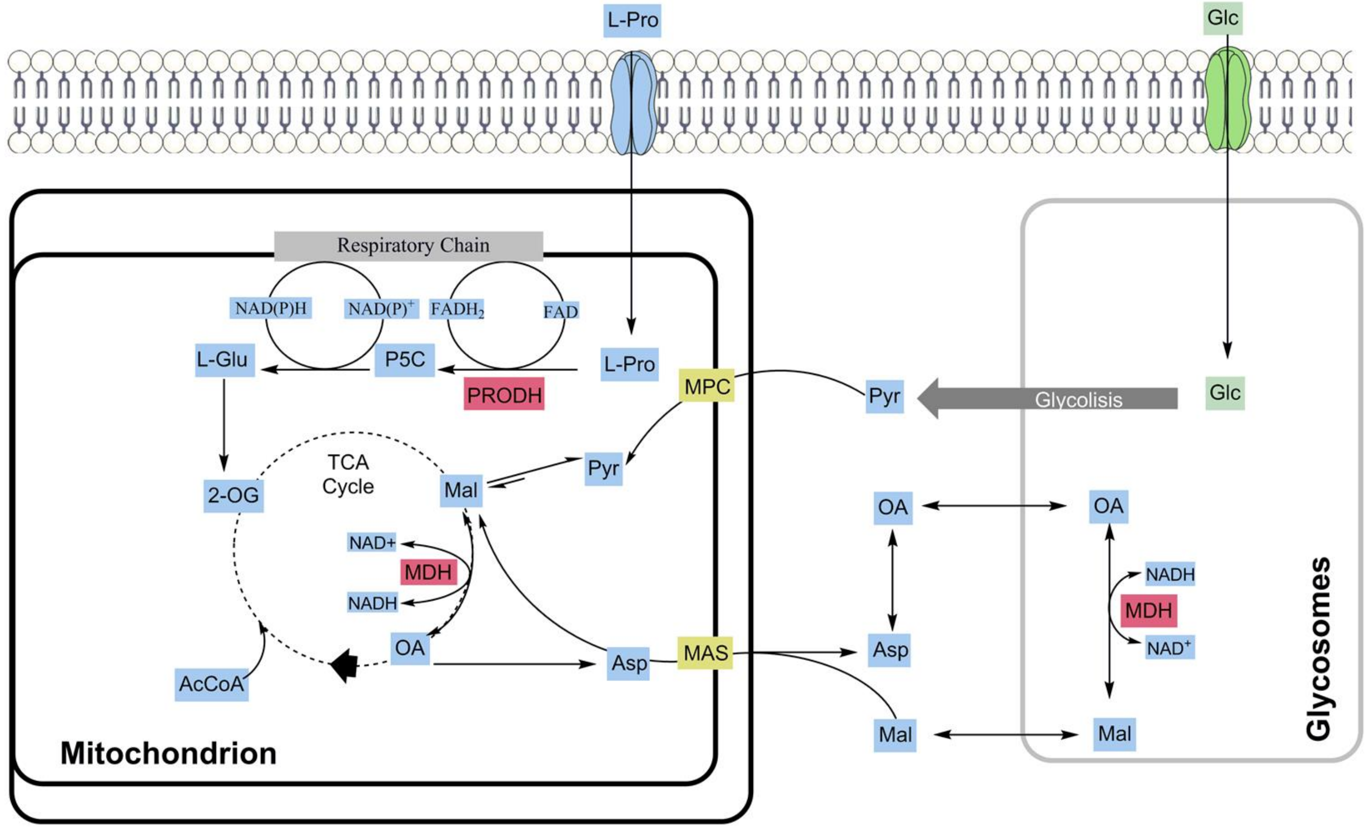

Supplement: Supplementary file 1 [file genes-09-00413-s001.zip › Supplementary information.pdf]
